# Identification of the Molecular Clockwork of the Oyster *Crassostrea gigas*

**DOI:** 10.1371/journal.pone.0169790

**Published:** 2017-01-10

**Authors:** Mickael Perrigault, Damien Tran

**Affiliations:** 1 University of Bordeaux, EPOC, UMR 5805, Arcachon, France; 2 CNRS, EPOC, UMR 5805, Arcachon, France; Karlsruher Institut fur Technologie, GERMANY

## Abstract

Molecular clock system constitutes the origin of biological rhythms that allow organisms to anticipate cyclic environmental changes and adapt their behavior and physiology. Components of the molecular clock are largely conserved across a broad range of species but appreciable diversity in clock structure and function is also present especially in invertebrates. The present work aimed at identify and characterize molecular clockwork components in relationship with the monitoring of valve activity behavior in the oyster *Crassostrea gigas*. Results provided the characterization of most of canonical clock gene including *clock*, *bmal*/*cycle*, *period*, *timeless*, vertebrate-type *cry*, *rev*-*erb*, *ror* as well as other members of the cryptochrome/photolyase family (*plant-like cry*, *6–4 photolyase*). Analyses of transcriptional variations of clock candidates in oysters exposed to light / dark regime and to constant darkness led to the generation of a putative and original clockwork model in *C*. *gigas*, intermediate of described systems in vertebrates and insects. This study is the first characterization of a mollusk clockwork. It constitutes essential bases to understand interactions of the different components of the molecular clock in *C*. *gigas* as well as the global mechanisms associated to the generation and the synchronization of biological rhythms in oysters.

## Introduction

Biological rhythms are a fundamental property of living organisms, driving behavior and physiology of animals, and maximizing their fitness by anticipating rhythmic changes in their environment [1]. Molecular origin of biological rhythms is composed of canonical “clock genes” organized around negative and positive transcriptional feedback loops [2–4]. Components of molecular clock systems have been exquisitely characterized in vertebrates, diverse insects and fungi [4]. From these studies, it appeared that some clock genes and interacting feedback loops are conserved across phyla [4 for review] but number of variations also existed and the divergence of molecular components and their function across phyla could be explain, in part, by gene duplication and loss [5]. However, few studies were focused on the molecular bases of clock systems in marine invertebrates [6,7] and more specifically in bivalves [8,9] whereas these organisms inhabited complex environments exposed to solar and lunar light entrainments (as terrestrial organisms) but also to tides [10,11].

The oyster *Crassostrea gigas* is an attractive organism to investigate biological rhythms and their molecular origin. For instance, this filter-feeder is world widely spread and represents high commercial interest. Development and application of technique of HFNI (High Frequency—Non Invasive) valvometry provided valuable information on oyster behavior and growth [12,13]. Previous works demonstrated the existence of a plastic dual circadian rhythm in oysters as well as robust tidal rhythms in the field [13,14]. Moreover, a cryptochrome belonging to the molecular clock was characterized in *C*. *gigas* and experiments demonstrated its transcriptional oscillation under tidal and circadian entrainments [9]. However, no other component of the molecular clockwork in oyster was characterized despite the large volume of molecular data as well as the available genome for this bivalve [15].

Objectives of this study were to identify and characterize clock genes involved in the generation and the synchronization of rhythms in the oyster *C*. *gigas*. Particularly, we identified homologs of *clock*, *bmal*, *period*, *timeless*, transcriptional repressor cryptochrome *cry2*, as well as other members of the cryptochrome/photolyase family. Further investigations on transcriptional variations of clock genes under light entrained regime and under constant darkness were coupled to the monitoring of valve activity behavior to unravel molecular clockwork of the oyster *C*. *gigas*.

## Materials and Methods

### Experimental procedures

Investigations on circadian behaviors and expression of clock genes were performed on 160 Pacific oysters *C*. *gigas* (diploid; 70 ± 1 mm shell length; mean ± SE) from oyster farm source ("Port du Rocher", La Teste de Buch, Arcachon bay, France, Lat. 44°38'N, Long. 1°7'O). Experiments were performed in an isolated room equipped with anti-vibrating benches to minimize external influences on animal behavior at the Marine Station of Arcachon (France) from February to April 2013. Oysters were split into two 150-L tanks and maintained in natural ([Chla] = 0.1 ± 0.07 μg·l^-1^) and oxygenated seawater of stable composition (T = 17.6 ± 0.1°C; pH = 7.9 ± 0.1; salinity = 34.9 ± 0.2 ‰; mean ± SD). Physical parameters of seawater were monitored with a R301 pH meter (Consort, Belgium) and a Cond 330 I conductivity probe (WTW, Germany). Following 10 days acclimation to lab conditions, oysters were maintained under L:D 10:14 cycle (light phase from ZT 0 to ZT 10 and dark phase from ZT10 to ZT 24) for 15 days. Oysters were sampled during light phases (ZT 1 in days 14 and 15, ZT 5, and ZT 9) and dark phases (ZT 11, ZT 15 and ZT 23 in days 13 and 14) starting on day 13 of L:D exposure (8 sampling times, S1 Fig). Remaining oysters were thereafter exposed to constant darkness for 15 additional days. Additional samplings were performed at circadian times CT 1 (day 14 and 15), CT 5, CT 9, CT 11, CT 15 and CT 23 (day 13 and 14) starting on day 13 of D:D exposure (8 sampling times). Synthetic diagram of experimental timeframe and sampling times was provided in S1 Fig. At each sampling time, gill tissue was individually dissected from 9 oysters under natural light during light phases or under dim red light during dark phases. Tissues were preserved in RNA later (Qiagen) at 4°C overnight and then transferred at -80°C until RNA extraction.

### Total RNA extraction and cDNA synthesis

Total RNA was extracted from individual samples using TRI^®^ Reagent (Invitrogen, Carlsbad, CA, USA). Total RNA quantity and quality were assessed by spectrophotometry (OD260, OD280) and 5 μg total RNA was individually submitted to reverse transcription using oligo dT17 and Moloney murine leukaemia virus (M-MLV) reverse transcriptase (Promega, Madison, WI, USA).

### Identification of clock candidates in *C*. *gigas*

Putative *C*. *gigas* clock sequences were identified through a local combination of tBLASTn and BLASTp searches of CDS, EST and genome [15] databases of *C*. *gigas* using Pfam conserved domains as well as homology with clock sequences identified in other organisms [6,16]. *CgCry1* was previously described and complete sequence was retrieved from Mat et al. [9]. Similarly, *ROR* and *Rev-Erb* homologs in oyster were retrieved from Vogeler et al. [17].

### Rapid amplification cDNA ends and sequence analysis of clock candidates

Full cDNA of candidates in *C*. *gigas*, including UTR, were obtained by RACE using specific primers (S1 Table) and methods described by Scotto Lavino et al [18,19]. Briefly, total RNA was extracted from gill tissue of oysters. Reverse transcription for the determination of 5' cDNA ends were performed with the SuperScript II reverse transcriptase (Invitrogen, USA) and RT primers (S1 Table). Similarly, 3' cDNA ends were amplified by PCR using reverse transcriptase and Qt primer. Sequences were amplified by PCR using Qo / specific Ro and Qo / specific Fo primer combinations. PCR products were thereafter used as template in nested PCR using Qi / specific Ri and Qi / specific Fi primer combinations (S1 Table) according to Scotto Lavino et al [18,19]. PCR products were separated on agarose gel and purified using Wizard^®^ SV gel and PCR clean up system (Promega, Madison, USA). Purified products were ligated into pGEM-T vector (Promega, MAdison, USA) and used to transform DH5α bacteria (Invitrogen, USA). Bacteria were cultured in Luria-Bertani broth medium containing 100 μg·ml^-1^ ampicillin and plasmids containing inserts were extracted and sequenced by extension from both ends using T7 and SP6 universal primers.

Complete cDNA candidates including UTR were mapped on *C*. *gigas* genome and presence of canonical E-box motif (CACGTG) was searched within a sequence of two kilobases upstream of each transcription start site for each gene. In presence of E-box motif, the site for each motif was annotated based on position upstream of the start-site according to Reitzel et al. [20].

The cDNA sequence and deduced amino acid sequence of candidates were analyzed and compared using BLAST algorithm (http://blast.ncbi.nlm.nih.gov/Blast.cgi) and the Expert Analysis System (http://www.expasy.ch/). Rooted phylogenetic trees of the different families of proteins were generated from sequence alignments by Maximum Likelihood method using Mega 6 software. Statistical confidence of inferred phylogenetic relationships were assessed by bootstraps of 1000 replicates.

### Real-time PCR analyses

Real time PCR reactions were performed on individual samples with Brillant III Ultra Fast SYBR Green QPCR Master Mix kit (Stratagene, USA) and a final concentration of 100 nM for each primer according to manufacturer’s instructions. Primer sets of clock candidates were designed from full cDNA sequence, excepted for *CgCry1*, *CgRev-Erb* and *CgROR* [9,17]; stability of the expression of glyceraldehyde-3-phosphate dehydrogenase (*GADPH*) and elongation factor (*EF1*) as reference genes were verified and *EF1* was used as reference gene (S1 Table). PCR efficiency (E) was assessed for each primer pair by determining the slope of standard curves obtained from serial dilution analysis of cDNA from different experimental samples. Reactions were initiated with activation of the DNA polymerase at 95°C for of 10 min followed by amplification of the target cDNA (40 cycles: denaturation at 95°C for 30 s, annealing at 58°C for 30s and extension at 72°C for 30 s). Reaction specificity was controlled using melting curve step from 95°C to 60°C (decrease of temperature of 0.5°C every 10 s).

The comparative Ct method (2^-ΔΔCt^ method, [21]) was used to determine transcript levels of candidates. Expression levels were normalized (ΔCt) in each sample using *EF1* (AB122066) sequence as housekeeping genes and ΔCt values from all samples were subtracted from the relative transcription level of candidates of each sample (ΔΔCt). Results are given as the mean (2^-ΔΔCt^) and standard deviation (n = 9).

### Data treatment and chronobiological analysis of the rhythm of valve behavior

Valve activity of oysters was continuously monitored during L:D and D:D exposures (*i*.*e*. 30 days). Fifteen oysters were equipped with HFNI (High Frequency—Non Invasive) valvometers to assess rhythms of oysters through variations of hourly valve opening duration according to Tran et al. [12,13]. Double-plotted actograms (each line represents 2 days) were produced with Chronos-Fit 1.06 [22]. Activity levels above the average of the day were represented by a black section, while values below the 24-h average were indicated by a white section. Chronobiological analyses were performed using the software Time Series Analysis Serie Cosinor 6.3 (http://www.euroestech.com). Data were processed and analyzed to validate a rhythm in oysters [23,24]. Briefly, the quality of the data set was assessed by controlling the absence of randomness using the autocorrelation diagram and the absence of a stationary character by a Partial Autocorrelation Function (PACF) calculation [25]. The periodicities in the recorded data were tested with the spectral method of the Lomb and Scargle periodogram [26]. These methods provide a threshold of probability (*p*-value = 0.95) defining the limit below which the signal can be considered as "noise". The confidence interval of the period was determined using the method of Halberg [27]. When a period was statistically validated, rhythmicity was modeled with the Cosinor model, which used a cosine function calculated by regression [28,29]. The model for a given period was written as: Y(t) = A.cos(2πt/τ + φ) + M + ϵ(t) where A was the amplitude, φ the acrophase, τ the period, M the mesor and ϵ the relative error [24]. Two tests provided the validation of the model: the elliptic test [29] had to be rejected and the probabilities (*p*-value) for the null amplitude hypothesis had to be lower than 0.05. For a set of data, many significant periodicities could exist. Identification of secondary periodicities was performed by the reinjection of residues from the previously calculated Cosinor model in a repeated procedure. Validation of significant rhythms were accomplished when the whole procedure, i.e. checks the quality of data, significant period by spectral analysis and the statistical validation of the Cosinor model, were achieved.

### Statistical analysis

Statistical analyses were performed using SigmaStat (Systat Software Inc., California, USA). ANOVA treatments that generated *p*-values below 0.05 were followed by a Holm-Sidak post-hoc test comparing different conditions. Non-parametric Kruskal-Wallis one-Way ANOVA on rank was performed whenever normality or homoscedasticity of data were not met. Correlations of transcript expressions between clock candidates were analyzed during LD and DD regimes using Spearman. Differences were considered statistically significant at *p* < 0.05.

## Results

### *C*. *gigas* clock sequences

Molecular approaches allowed the identification of homologs for most of the known invertebrates and mammals clock genes in the oyster *C*. *gigas* (S2 Fig). Some of which were previously characterized such as *CgCry1* [9] or identified such as *CgRev-erb* and *CgROR* [17]. For instance, Voleger et al. [17] analyzed nuclear receptor genes from the genome of *C*. *gigas* and demonstrated the presence of homologs of NR1F and NR1D that clustered with human *ror* and *Rev-erb* genes respectively. Full sequences of clock candidates (*CgClock*, *CgPeriod*, *CgCry2*, *CgBmal*, *CgTim*) and other cryptochrome/photolyase homologs (*Cg6-4photolyase*, *CgpCry*) were obtained by RACE, deposited into the GenBank database (see S2 Table for accession numbers) and deduced amino acid sequences were used for further analyses of protein structure and phylogenetic relationships (see Methods). Additionally, full mRNA sequences including UTR were mapped in the genome of *C*. *gigas* [15] to identify E-box motifs. In mammals and insects, Clock:Cycle/Bmal heterodimers regulate transcription of downstream genes by binding to E-box motifs [30,31]. E-box motif was designed based on consensus sequences [32,33]. Analyses demonstrated the presence of E-box motifs in the genome of *C*. *gigas* near the transcription initiation sites of *CgCry2* (-36 bp), *CgCry1* (-274 bp) and *CgPeriod* (-86 bp) but no identical match were found for *Cg6-4photolyase*, *CgBmal*, *CgClock* and *CgpCry*. Absence of complete sequences for *CgROR* and *CgRev-erb* (retrieved from another study) did not allow the search of motifs and mapping of *CgTim* 5’UTR on *C*. *gigas* genome was unsuccessful.

**Fig 1:**
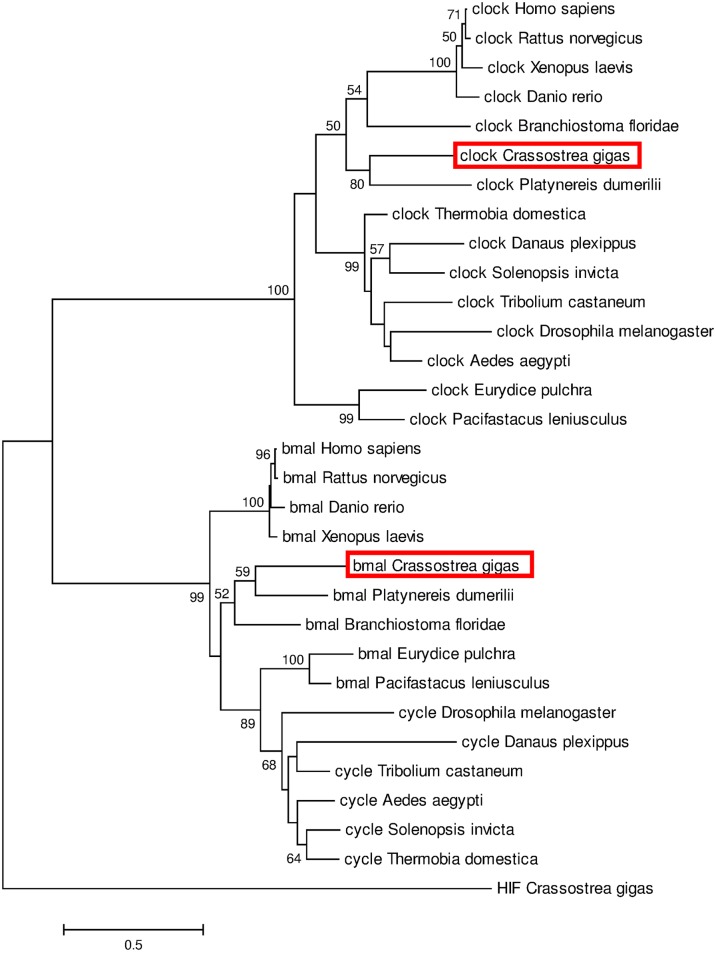
Phylogenetic analysis based on clock and cycle/Bmal sequences. The tree was generated by Maximum Likelihood method using Mega 6 program and based on the multiple alignments performed with Clustal Omega. Percentage of bootstraps based on 1,000 replicates were indicated with only values > 50%. Hypoxia-inducible factor (HIF) from *C*. *gigas* was used as outgroup to root the tree. See S2 Table for sequence details and accession numbers.

Clock and Cycle/Bmal are PAS-bHLH transcription factors. *C*. *gigas* ortholog of Cycle/Bmal was named CgBMAL based on its clustering with other Bmal sequences (Fig 1). Both CgCLOCK and CgBMALl contains specific features of the family with highly conserved PAS-A, PAS-B, PAC and bHLH domains as well as NLS and NES signals [34,35]. Moreover, phylogenetic analyses demonstrated a good discrimination between Clock and Cycle/Bmal proteins with, globally, a clustering of CgCLOCK with vertebrate clock proteins and a closer distance of CgBMAL with Cycle/Bmal protein from arthropods than vertebrate ones. More specifically, close phylogenetic relationships of CgCLOCK and CgBMAL were observed with their counterpart from the marine worm *Platynereis dumerilii* (Fig 1).

**Fig 2:**
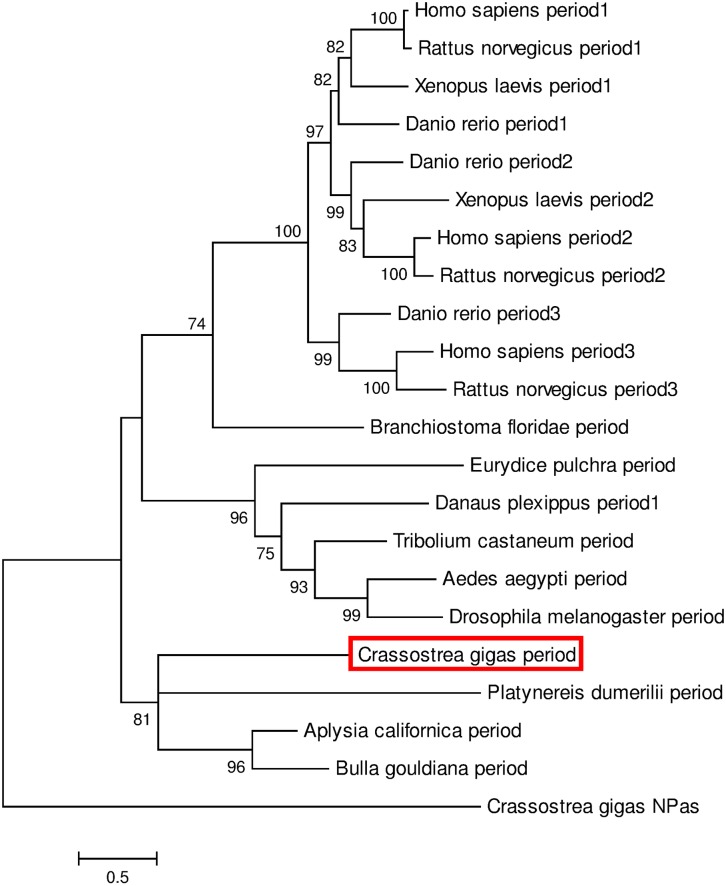
Phylogenetic analysis based on period sequences. The tree was generated by Maximum Likelihood method using Mega 6 program and based on the multiple alignments performed with Clustal Omega. Percentage of bootstraps based on 1,000 replicates were indicated with only values > 50%. Neuronal PAS domain-containing protein (NPas) from *C*. *gigas* was used as outgroup to root the tree. See S2 Table for sequence details and accession numbers.

Complete sequence of *CgPeriod* (4216 bp) revealed an open reading frame of 3945 bp encoding 1315 amino acids exhibiting specific characteristics of PERIOD proteins such as the two tandemly organized PAS domains [35–37] (S2 Fig). Phylogenetic analysis of PERIOD proteins from vertebrates and invertebrates demonstrated a clustering of CgPERIOD with PERIOD proteins from other marine mollusks and annelids compared to vertebrate and arthropod counterparts (Fig 2).

**Fig 3:**
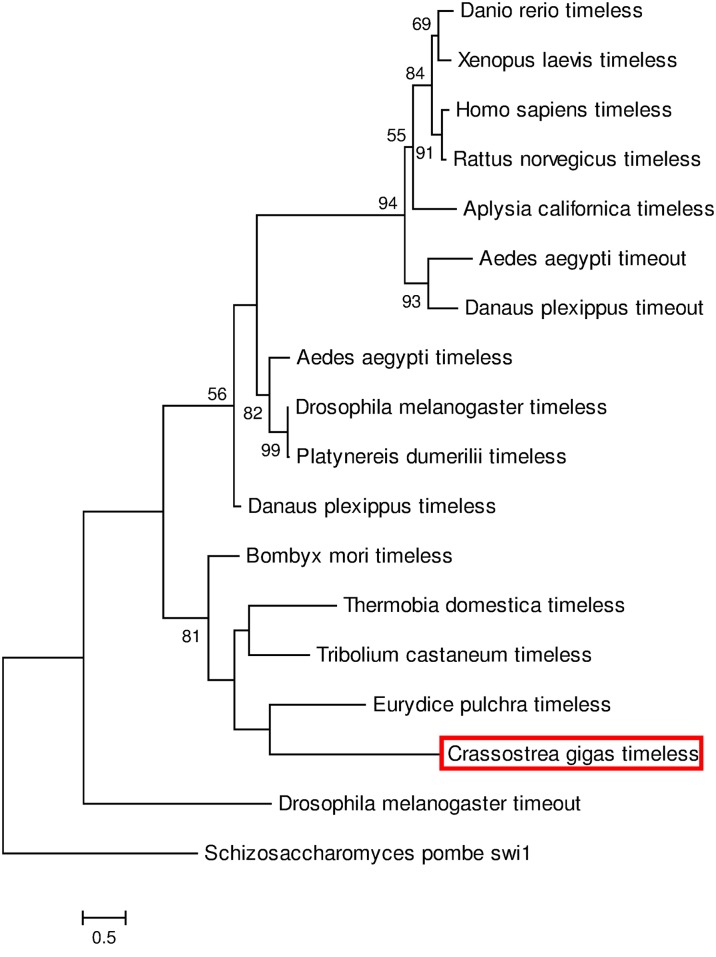
Phylogenetic analysis based on timeless sequences. The tree was generated by Maximum Likelihood method using Mega 6 program and based on the multiple alignments performed with Clustal Omega. Percentage of bootstraps based on 1,000 replicates were indicated with only values > 50%. Swi1 from *Schizosaccharomyces*. *pombe* was used as outgroup to root the tree. See S2 Table for sequence details and accession numbers.

Sequence belonging to the Timeless/Timeout family was also identified in *C*. *gigas*. *CgTim* (4750 bp) was composed of an open reading frame of 2802 bp corresponding to 934 amino acids. Surprisingly, CgTIM was not phylogenitically related to the annelid Timeless (*P*. *dumerilii*) but clustered with Timeless proteins from insects and crustaceans (Fig 3).

**Fig 4:**
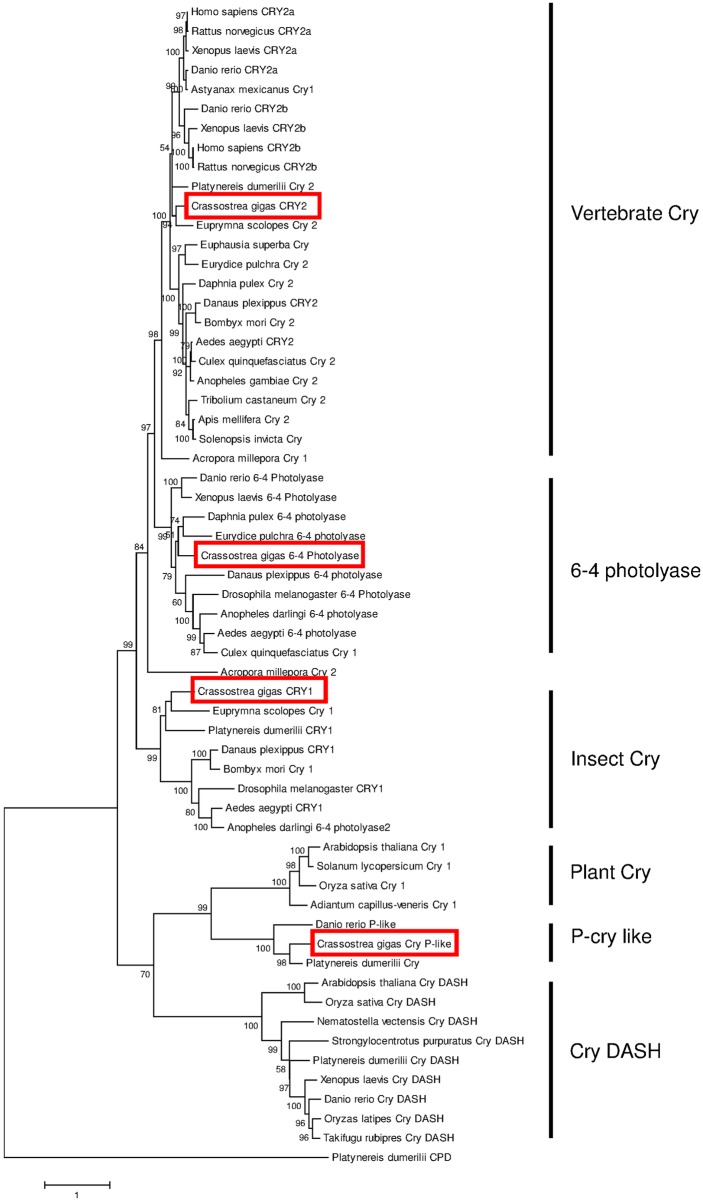
Phylogenetic analysis based on cryptochrome / photolyase sequences. The tree was generated by Maximum Likelihood method using Mega 6 program and based on the multiple alignments performed with Clustal Omega. Percentage of bootstraps based on 1,000 replicates were indicated with only values > 50%. CPD photolyase (CPD) from *P*. *dumerilii* was used as outgroup to root the tree. See S2 Table for sequence details and accession numbers.

**Fig 5:**
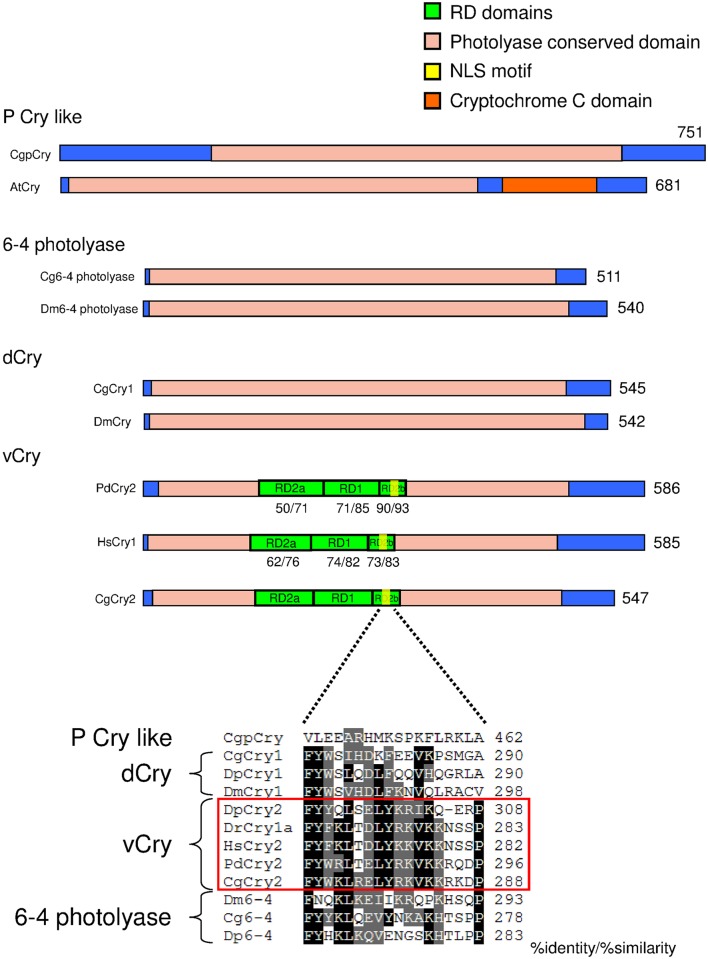
Schematic representation of putative functional domains and motifs in photolyase / cryptochrome proteins in oyster, *Arabidopsis thaliana* (AtCRY), fruit fly (Dm6-4PHOTOLYASE and DmCRY), marine worm (*Platynereis dumerilii*, PdCRY2) and human (HsCRY1). The number at the end of each diagram indicated protein size in amino acid residues. Numbers below domains indicated sequence identity / similarity with oyster ortholog. Inset showed multiple sequence alignments of a putative nuclear localization signal (NLS) in the RD2b domain and sequences referred to oyster (CgpCRY, CgCRY1, CgCRY2, Cg6-4PHOTOLYASE), human (HsCRY2), fruit fly (DmCRY1 and Dm6-4), zebrafish *Danio rerio* (DrCRY1a), butterfly *Danaus plexippus* (DpCRY1, DpCRY2 and *Dp6-4*) and marine worm *P*. *dumerilii* (PdCRY2).

Molecular approaches led to the characterization of 3 sequences belonging to the Cryptochrome/Photolyase family, in addition to the previously characterized *CgCry1* [9]. All candidates contained DNA photolyase and FAD binding domains. Phylogenetic analysis of Cryptochrome/photolyase proteins from vertebrates and invertebrates generated a specific dichotomy of the cryptochromes with the presence of several groups previously identified by Oliveri et al. [16]: 6–4 photolyase, vertebrate-type Cry (*vcry* or *cry2*), insect-type Cry (*dcry* or *cry1*), plant-like cry and cry DASH. Within each sub group, oyster sequences were closely related to *P*. *dumerilii* homologs when both sequences were represented (Fig 4). Further analysis of CRYPTOCHROME/PHOTOLYASE proteins organization in *C*. *gigas* demonstrated the structural homology of Cg6-4PHOTOLYASE and CgCRY1 with their counterpart in *Drosophila*. Similarly, CgCRY2 was composed of highly conserved repressor domains (RD) involved in the function of transcriptional repression of CLOCK:CYCLE/BMAL heterodimer [34,35] (Fig 5). More specifically, insert of the Fig 5 showed a high amino acid conservation of CgCRY2 in the sequence alignment corresponding to the nuclear localization signal (NLS) within the RD2b region that is necessary for mammalian-type cry nuclear localization and subsequent repression of CLOCK:CYCLE/BMAL transcription complex. CgPCRY differed from other plant cryptochromes and more generally from CRYPTOCHROME/PHOTOLYASE proteins by the presence of an extended N-terminal fragment before the conserved photolyase/FAD domain (Fig 5).

### Expression of clock genes

**Fig 6:**
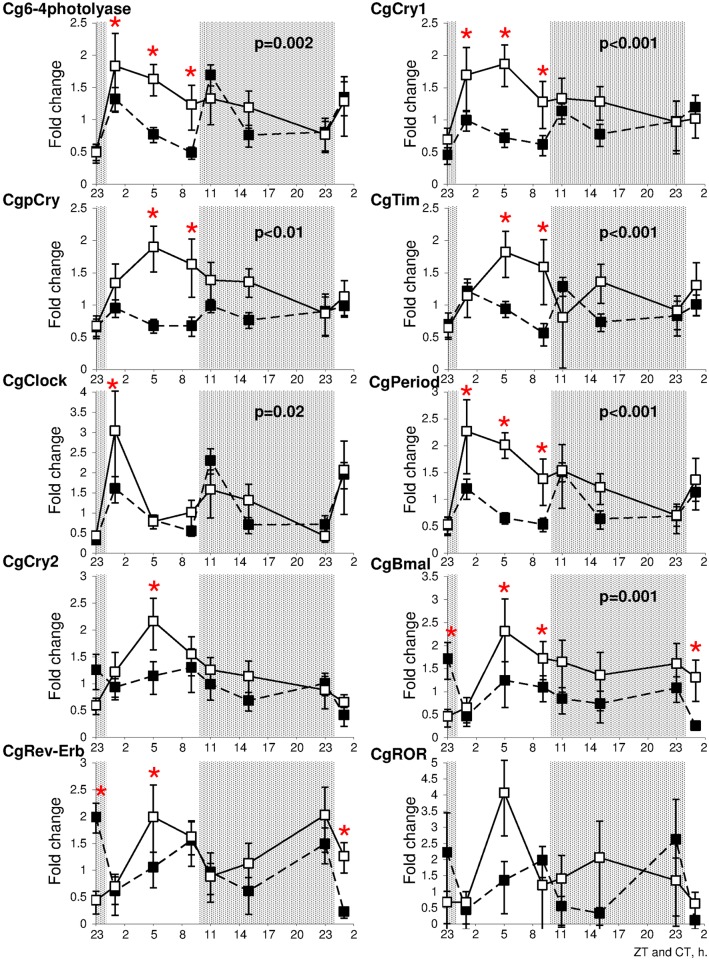
Variation of expression levels of clock genes. Relative transcription levels (RQ, mean ± SEM, n = 9 oysters) of *Cg6-4photolyase*, *CgCry1*, *CgpCry*, *CgCry2*, *CgClock*, *CgBmal*, *CgPeriod*, *CgTim*, *CgRev-Erb* and *CgROR* RNA in gill tissues of oysters exposed for 26 h to L:D 10:14 (□) and for 26 h to constant darkness (■). Gray areas referred to scotophase during L:D cycles. Significant differences at *p* < 0.05 between light regimes are indicated and asterisks denote significant time-specific differences between light regime treatments.

Investigations of transcriptional variations of clock candidates and Cryptochrome/Photolyase family members in *C*. *gigas* under L:D (10:14) and D:D regimes led to the observation of different profiles. Expression for most of candidates peaks during the photophase under L:D regime with a maximum expression at ZT 1 for *Cg6-4photolyase*, *CgClock* and *CgPeriod* whereas *CgCry1*, *Cgpcry*, *CgTim*, *CgCry2* and *CgBmal* tended to peak at ZT 5 (Fig 6). It is interesting to note that *CgRev-erb* peaks twice, during the photophase (ZT 5) and during the scotophase (ZT 23). Exposure of oyster to constant darkness was associated with significant modulation of gene expressions (Fig 6). For instance, *Cg6-4photolyase*, *CgCry1*, *CgpCry*, *CgTim CgClock* and *CgPeriod* tended to increase transcription levels during transitional illumination regime corresponding to light on/off and despite the absence of light shifting under D:D regime (CT 23–1 and CT 9–11). On the contrary to these profiles, *CgCry2*, *CgBmal*, *CgRev-erb* and *CgROR* exhibited opposite trends under D:D regime with a decrease of expression during the times associated to transitional light regime under L:D (Fig 6).

Correlation analyses at individual level revealed the presence of two groups of genes with a positively correlated transcription level during L:D and D:D regimes (Tables 1 and 2). For instance, transcription levels of *Cg6-4photolyase*, *CgCry1*, *CgpCry*, *CgTim*, *CgClock* and *CgPeriod* followed similar trends of variations with ρ ranging from 0.4 to 0.944 (*p* < 0.001, Table 1). Similarly, expression of *CgCry2*, *CgBmal CgRev-erb* and *CgROR* followed similar modulations with ρ ranging from 0.273 to 0.891 (*p* < 0.02, Table 2). However, correlation modifications associated to changes of light regimes were observed. During L:D regime, *CgCry2* was positively correlated to the group composed of *CgPeriod* and *CgClock* (*p* < 0.02, ρ 0.264–0.37) whereas D:D regime abolished these relationships. Additionally, D:D regime was associated to negative correlations of *Cgclock*, *CgPeriod*, *CgCry1* and *Cg6-4photolyase* with *CgBmal* and *CgRev-erb* (*p* < 0.04, Table 2).

**Table 1:** Spearman analysis of clock gene expression under L:D regime (n = 72 oysters).

	*CgCry1*	*CgpCry*	*CgTim*	*CgClock*	*CgPeriod*	*CgCry2*	*CgBmal*	*CgRev-Erb*	*CgROR*
*Cg6-4* *photolyase*	*** 0.872	*** 0.852	*** 0.83	*** 0.869	*** 0.933	** 0.325			
*CgCry1*		*** 0.944	*** 0.81	*** 0.652	*** 0.83	** 0.37			
*CgpCry*			*** 0.836	*** 0.649	*** 0.865	** 0.329			
*CgTim*				*** 0.694	*** 0.846	** 0.323			
*CgClock*					*** 0.857	* 0.264			
*CgPeriod*						** 0.334			
*CgCry2*							*** 0.492	*** 0.412	*** 0.4
*CgBmal*								*** 0.844	*** 0.614
*CgRev-Erb*									*** 0.508

*p* < 0.05 *,

*p* < 0.01 **,

*p* < 0.001 ***

Values indicate correlation coefficients (ρ)

**Table 2:** Spearman analysis of clock gene expression under D:D regime (n = 72 oysters).

	*CgCry1*	*CgpCry*	*CgTim*	*CgClock*	*CgPeriod*	*CgCry2*	*CgBmal*	*CgRev-Erb*	*CgROR*
*Cg6-4* *photolyase*	*** 0.805	*** 0.758	*** 0.806	*** 0.888	*** 0.872		** -0.308	* -0.264	** -0.328
*CgCry1*		*** 0.891	*** 0.751	*** 0.709	*** 0.829		* -0.287	* -0.257	
*CgpCry*			*** 0.817	*** 0.671	*** 0.851				
*CgTim*				*** 0.777	*** 0.838				
*CgClock*					*** 0.856		** -0.316	* -0.298	** -0.33
*CgPeriod*							* -0.273	* -0.245	* -0.244
*CgCry2*							*** 0.592	*** 0.623	* 0.273
*CgBmal*								*** 0.849	*** 0.435
*CgRev-Erb*									*** 0.504

*p* < 0.05 *,

*p* < 0.01 **,

*p* < 0.001 ***

Values indicate correlation coefficients (ρ)

### *C*. *gigas* valve activity

**Fig 7:**
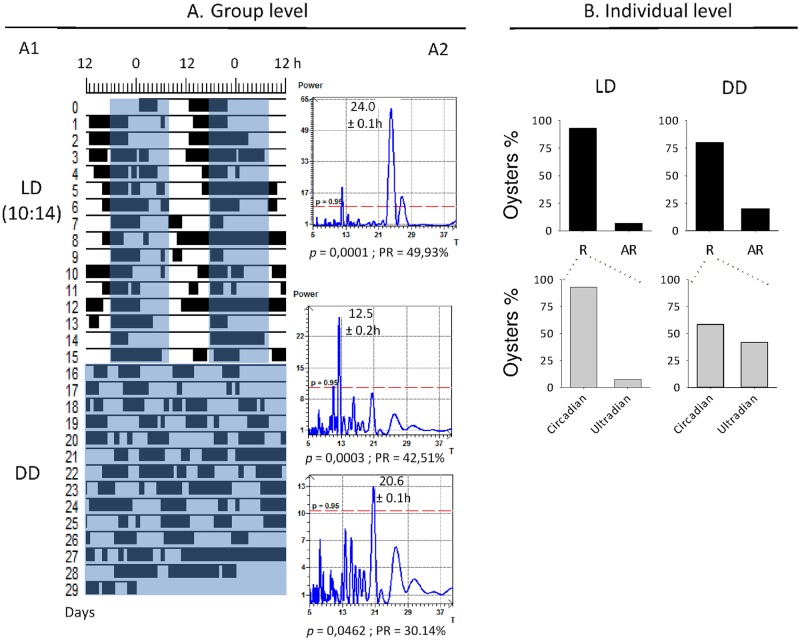
Oysters expressed circadian cycles of valve activity behavior. A. Group level analysis. Left panel (A1): Double-plotted actograms of mean hourly opening duration (%) of the group (n = 15 oysters) submitted to 15 days of L:D (10:14) and 15 days of D:D. Right panel (A2): Period determined by spectral analysis (Lomb and Scargle periodogram; dotted line for *p*-value = 0.05) and percent rhythm (PR) of the Cosinor model. B. Individual analysis. Distribution of rhythmic (R) and arrhythmic (AR) oysters among LD and DD conditions. Details of periods in rhythmic oysters (circadian: 20-28h and ultradian <20h) are provided.

Oyster subjected to a L:D (10:14) regime for 15 days exhibited a daily valve activity, with higher opening activity at the end of the photophase and at the beginning of the scotophase (Fig 7). Spectral analysis of the population revealed an oscillation of oyster behavior at a period of 24.0 h. Further analyses at individual level indicated that 93.3% of oysters exhibited rhythmicity with 92.9% oysters presenting circadian behavior of 24 h. Following exposure to constant darkness for 15 additional days, several behavioral modifications were noticed. For instance, circadian rhythmicity was still observed at a population level (period of 20.6 h) but ultradian period of 12.5 h was also detected. At the individual level, the proportion of oysters exhibiting rhythmicity dropped to 80% and within these animals, only 58.3% had a circadian behavior under constant darkness (Fig 7).

## Discussion

This study led to the characterization of core genes involved in the clock system of the oyster *C*. *gigas* as well as several members of the Cryptochrome/Photolyase family. Molecular characterization was coupled to investigations of transcriptional variations of candidates in gill tissues under different light regimes as well as behavior analyses of oyster through the application of HFNI valvometry technique [13]. Gill tissue was selected for its physiological role (nutrition, respiration) in relationship to valve activity.

Several members of the Cryptochrome/Photolyase family characterized in this study were not directly related to the clock system in *C*. *gigas* according to their phylogenetic position and previous works [38,39]. *Cg6-4photolyase* belonged to enzymes that catalyze light-dependent DNA repair [38–40]. Reactivity of 6–4 photolyase to the light was previously demonstrated [16] and observed profiles of *Cg6-4photolyase* under D:D regime in our experiment could be due to their affiliation as clock-controlled genes (CCG) [4]. However, some members of the Cryptochrome/Photolyase family were shown to exhibit 6–4 photoproduct repair activity and could act as transcriptional repressor in the circadian clock system [41]. Molecular approaches also provided the identification of CgPCRY which belonged to a cluster of proteins of unknown function proposed by Oliveri et al. [16] and closely related to plant cry, well known as light sensor and light-dependent circadian regulators in plants [42,43]. Homologs of pCry-like sequences were identified in marine animals including *P*. *dumerilii* and *Danio rerio* and exhibited regular circadian changes of expression, but were absent in *Drosophila* and mammals [16]. Despite the lack of information on pCry-like molecules, it is suggested that pCry like class should have been present in the last common eukaryotic ancestral organism that populated the oceans and it has been extensively lost in many metazoan lineages. Moreover, pCry-like protein might be a possible candidate as a circadian sensor in marine animals [16]. We should note that CgPCRY exhibited specific feature characterized by an extended N-terminal region compared to other Cryptochrome/Photolyase members [44].

**Fig 8:**
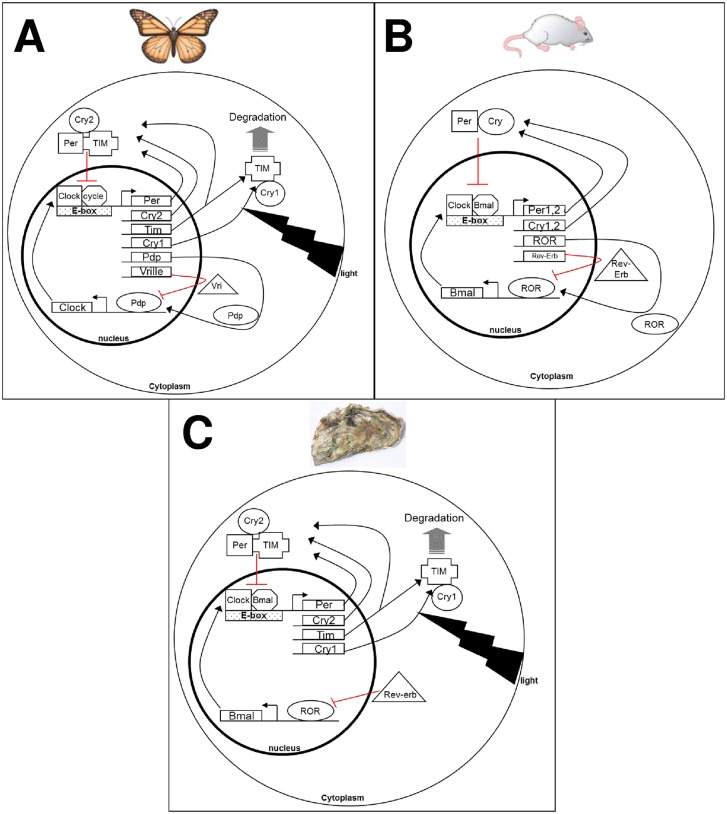
Schematic representation of the hypothetic molecular clockwork of the oyster *Crassostrea gigas*. Simplified molecular oscillators from butterfly (A) and vertebrate (mouse) (B) were presented in comparison to the putative model in *C*. *gigas* (C). Red lines indicated inhibition. Adapted from Chaves et al. [38] and Emery and Reppert [49].

Biological rhythms and their molecular bases were intensively studied in terrestrial organisms but little is known in marine invertebrate species. Biological rhythms had a molecular origin based on several interlocked transcriptional and post-translational auto-regulatory feedback loops controlling the molecular clock. A common feature among organisms was characterized by the formation of the heterodimer CLOCK:CYCLE/BMAL which, upon binding on E-box element, activated the transcription of genes under its control [2,3,45]. Genes harboring E-box motifs and under the control of CLOCK:CYCLE/BMAL complex included vertebrate type cryptochromes (*vcry*, transcriptional repressors) and *period* genes. However, differences were observed according to phyla and species. For instance, in vertebrate, CLOCK:CYCLE/BMAL heterodimer activated the transcription of *period* and *vcry* genes which formed complexes and repressed CLOCK:BMAL transactivation (Fig 8B). In *drosophila* and silkmolk, CLOCK:CYCLE complex controlled the transcription of insect type cryptochrome (*dcry*, photoreceptors), *period* and *timeless*. In this case, period and timeless formed complexes that interacted with CLOCK:CYCLE heterodimer [46,47]. In addition to these models, intermediate systems were observed in several insects including the monarch butterfly (*Danaus plexippus*), the Chinese oak silkworm *Bombix mori* and the mosquito *Anopheles gambiae* (Fig 8A) [48]. Similar clock gene diversities and functions were found in the marine worm *Platynereis dumerilii* [6]. Results of the present study on *C*. *gigas* presented also similar diversity of clock genes and phylogenetic analyses demonstrated a close relationship of clock sequences between *P*. *dumerilii* and *C*. *gigas*. Moreover, correlation analyses as well as the identification of E-box motifs were in accordance to the molecular clockwork organization of the monarch butterfly (Fig 8A).

However, Rev-erb and ROR orthologs were also identified in *C*. *gigas*. Expression of these genes was correlated to the expression of *CgBmal* in our experiments. Such results tended to support the existence of an additional regulating loop on *Bmal* as in vertebrates (Fig 8B). To date, no other homologs of Rev-erb and ROR were identified in invertebrates, excepted in the mussel *Mytilus californianus* where ROR homolog was found and exhibited significant circadian rhythmicity [8]. Attempts to identify Rev-erb and ROR homologs in the cnidarian *Nematostella vectensis* were unsuccessful [50]. It is also possible that regulation loop involving clock and homologs of pdp and vrille as in insect molecular clockwork, could exist in *C*. *gigas* since homologs of vrille and pdp were identified in *P*. *dumerilii* [6]. However previous works suggested an intermediate phylogenetic position of PDP homolog in *C*. *gigas* [34].

Surprisingly, transcriptional expression of *CgCry2* was not correlated to the expression of *CgPeriod* and *CgTim* in oysters under D:D regime despite the presence of E-box motif on its genomic sequence and in opposition to oysters exposed to L:D regime (Tables 1 and 2). However, correlation coefficients of *CgCry2* with other genes were globally low under L:D and D:D conditions (0.264 < ρ < 0.623) and discrepancies of results associated to the absence of zeitgeber (light regime) could suggest the presence of an additional regulation mechanism of *CgCry2*, independent from the regulation associated to the complex CLOCK:BMAL. Many aspects of molecular clockworks in animals, especially in marine organisms, still needed to be unraveled and were highlighted by the recent discovery of a new regulation loop in vertebrates [51]. Moreover, exposure of oyster to D:D regime was associated with an increase of the proportion of oysters exhibiting ultradian period but the relationships between oyster behaviors and gene expressions appeared complex to explain since QPCR results were performed on gills samples from oysters exhibiting circadian and ultradian rhythms and could therefore mask or alter observed profiles of gene expression. Further investigations need to be engaged to decipher the regulation and interaction of clock genes such as *Cgcry2*, as well as the global molecular clockwork of *C*. *gigas* in relationship with behavior activities. It was also tempting to associate observed ultradian periods (12.5 h) as well as two peaks observed in several expression profiles of clock genes during D:D exposure with tidal rhythms that oysters exhibited in the field [13]. In lab-controlled environment, simulated tidal entrainment induced an oscillation of *CgCry1* transcripts at a tidal frequency [9]. However, tidal rhythms in oysters were never previously observed in free running conditions [14,52]. Several theories were proposed to explained biological rhythms of marine organisms exposed to solar cycles and tides [53]. Recent studies indicated the existence of different molecular mechanisms associated to circatidal / circalunar and circadian rhythms but, to date, no specific gene was identified in association with tidal rhythms [6,7].

Mat et al. [14] demonstrated that *C*. *gigas* exhibited a dual circadian rhythm with a nocturnal activity mainly in autumn and winter and shifted to diurnal activity in spring and summer. This dualism was observed in the present study with an intermediate stage of oyster valve activity in relationship with the time of experiments (end of winter, beginning of spring). Interestingly, expression of *CgCry1* under L:D regime also followed a shift of expression profile according to the time of the year and could initiate observed behavioral shift. Tran et al. [54] performed experiments on oysters during winter time and observed nocturnal activities associated with higher expression of *CgCry1* in oyster gills during the scotophase whereas our experiments, performed on oyster experiencing progressive shift to diurnal activities, were associated with higher expression of *Cgcry1* in oyster gills during the photophase.

Lack of extensive studies in chronobiological organization and functions in bivalves are also highlighted by the absence of well-defined central system. It was suggested that some organisms could be composed of oscillators located in different tissues and coordinated in absence of central neural system but it is likely not the case in *C*. *gigas* since Ellis and Kempf [55] described several ganglia characterizing central nervous system as well as neuronal connections between these ganglia and several tissues in oyster larvae. For instance, gill tissues used in this study to investigate transcriptional variations of clock candidates are likely a peripherical clock of *C*. *gigas*. Previous studies demonstrated that biological rhythms are tissue-dependent [56]. Period gene from *Bulla gouldiana* exhibited no rhythmic expression in peripherical tissues [36]. Moreover, clock components such as *dcry* in *drosophila* could act as a light sensor as well as light-independent clock component of peripherical oscillators according to the tissue [48,57]. Previous published results on the characterization of *Cgcry1* demonstrated that under similar experimental conditions, oscillation of *Cgcry1* transcripts was absent in smooth muscle and expression profiles of *CgCry1* in striated muscle were significantly different from our results in gill tissues [9].

In conclusion, this study provided for the first time in a mollusk bivalve, the identification and the characterization of several genes involved in the molecular clock system of the oyster *C*. *gigas* and proposed a putative and original clockwork model intermediate of described systems in vertebrates, annelids and insects (Fig 8C). However, some genes (such as *CgpCry*) and results also raised numerous questions about the organization of the clock system and the function of the different components in relationship with the behavior of oysters which inhabited complex environments exposed to multiple zeitgebers. Further researches need to be pursued to unravel the complexity of the molecular clockwork of the oyster *C*. *gigas*.

## Supporting Information

S1 Fig Schematic representation of experimental timeframe and sampling times. (TIF)

S2 Fig Details of characterized clock genes. Nucleotide and deduced amino acid sequences of *Cg6-4photolyase*, *CgpCry*, *CgCry2*, *CgClock*, *CgBmal*, *CgPeriod* and *CgTim* in *C*. *gigas*. Numbering along the left margin. The start and stop codons are marked in bold. Sequences were deposited (NCBI) and accession numbers are indicated in S2 Table.
(DOC)

S1 Table list of primers. (DOC)

S2 Table details and accession numbers of sequences used in phylogenetic analysis. (DOC)
