# Supplementary figures and images for: Identification of the Molecular Clockwork of the Oyster Crassostrea gigas
Source: PLoS One. 2017 Jan 10;12(1):e0169790. doi: 10.1371/journal.pone.0169790 (PMC5224872; doi:10.1371/journal.pone.0169790)

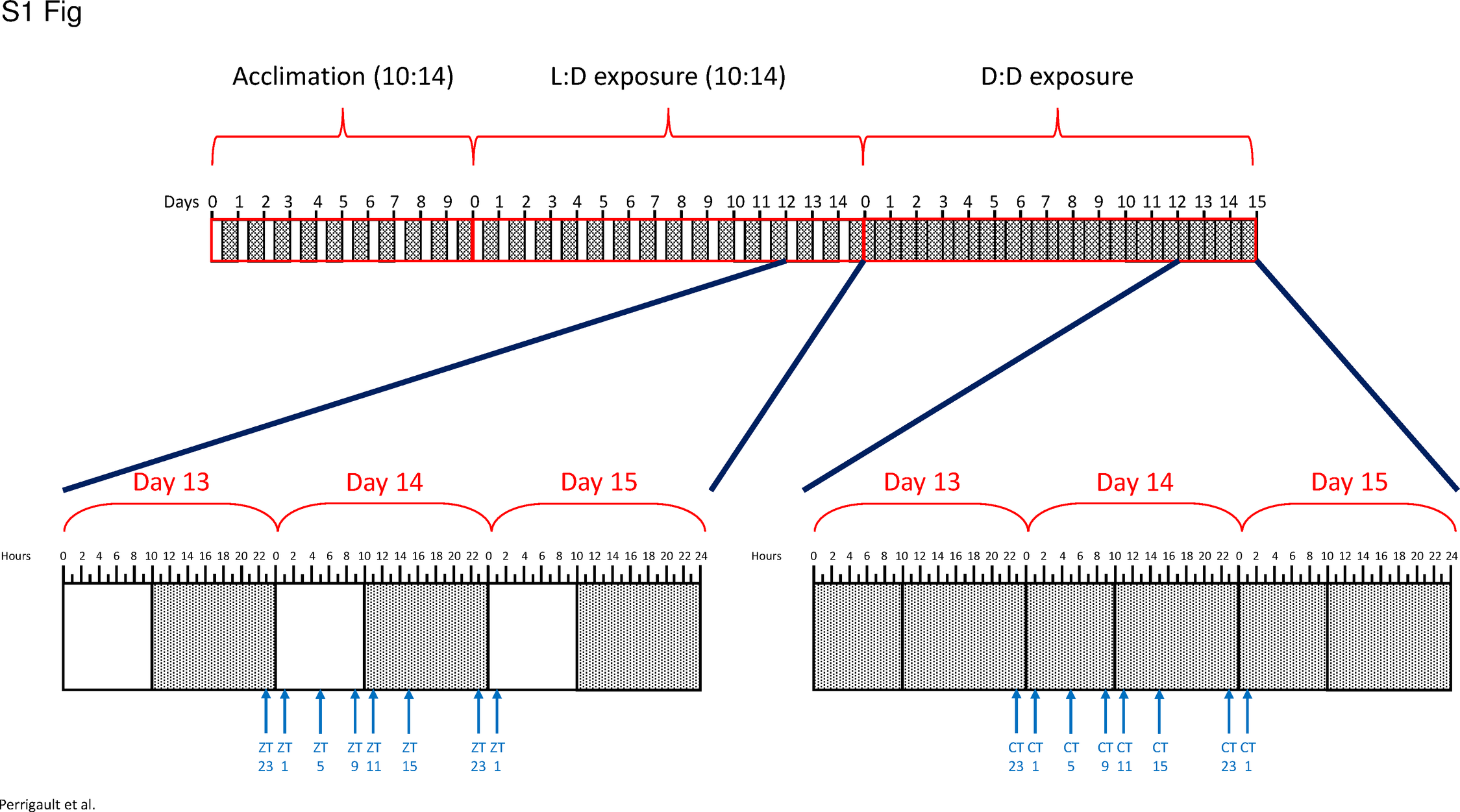

Supplement: S1 Fig — (TIF) [file pone.0169790.s001.tif]
