# Supplementary material for: Identification of the Molecular Clockwork of the Oyster Crassostrea gigas
Source: PLoS One. 2017 Jan 10;12(1):e0169790. doi: 10.1371/journal.pone.0169790 (PMC5224872; doi:10.1371/journal.pone.0169790)
Supplement: S2 Fig — Nucleotide and deduced amino acid sequences of Cg6-4photolyase, CgpCry, CgCry2, CgClock, CgBmal, CgPeriod and CgTim in C. gigas. Numbering along the left margin. The start and stop codons are marked in bold. Sequences were deposited (NCBI) and accession numbers are indicated in S2 Table. (DOC) [file pone.0169790.s002.doc]

Figure S1

Nucleotide and deduced amino acid sequences of *6-4 photolyase*, *Clock*, *Cry2*, *Bmal*, *Period*, *Timeless* and *P like Cry* in *C. gigas*. Numbering along the left margin. Start and stop codons are indicated in bold.

>Cg6-4photolyase

1 tacgtaacgaaaattttggttgcgtgcgtatttatctaaatatcaatacatgttcaattgaataga**ATG**AAAACT

1 M K T

76 TGTGCAATTCACTGGTTTCGTAAAGGATTGAGACTGCATGATAATCCAGCACTTCAGGCAGCGTGCAAAGTTGCT

4 C A I H W F R K G L R L H D N P A L Q A A C K V A

151 GACGACGTGAAGCCAGTATTTATATTGGACCCTTGGTTTGCCAACAATGCTAATGTAGGCGTAAATAGGTGGAGA

29 D D V K P V F I L D P W F A N N A N V G V N R W R

226 TTCCTCTTGCAGACGTTACAAAACTTAGATGAAAACTTGAAAAAAATAAATTCACGATTATACATTATAAAAGGA

54 F L L Q T L Q N L D E N L K K I N S R L Y I I K G

301 AAGCCGGCAGATGTATTTCCAAAGTTATTTAAAAATTGGGGAGTGAGCCATCTGACTTTTGAAGAAGACATAGAA

79 K P A D V F P K L F K N W G V S H L T F E E D I E

376 CCTTATGCTTTAACGAGAGATTCTGAGATTAAGAAGTTAGCAGATGAACATAATGTAAAGGTGACATCTTGTGTC

104 P Y A L T R D S E I K K L A D E H N V K V T S C V

451 TCCCACACATTATTTGATCCACAAAGAATAATCTCAAAGAATGGTGGCAAGGCTCCCCTAACATACCAACGTCTT

129 S H T L F D P Q R I I S K N G G K A P L T Y Q R L

526 CAGACAGTGTTGTCAAGCCTTGGTTCTCCCCCCAAACCAGTAGACAGCCCATCAGAATGTAAGACAAAGACAGAA

154 Q T V L S S L G S P P K P V D S P S E C K T K T E

601 TCAGACCATGACAAGAAATACGGTGTTCCCTCCCTGGAGGATTTGGGGAAATCAGAGAAAGAATGTGGTCCCCTT

179 S D H D K K Y G V P S L E D L G K S E K E C G P L

676 CTGTTTCCGGGAGGAGAAACAGAGGCCCTGAGGAGACTGGAGAGCATGATGGGAAAAAAAAACTGGGTTTGCACA

204 L F P G G E T E A L R R L E S M M G K K N W V C T

751 TTTGAGAAACCTAAGACTGCCCCCAACAGTCTGGAACCCAGTACAACAGTACTGAGTCCTTACCTGAAGTTTGGC

229 F E K P K T A P N S L E P S T T V L S P Y L K F G

826 TGTCTCTCTCCCAGAATGTTCTACTACAAGCTACAAGAGGTCTATAATAAAGCCAAGCATACCTCGCCCCCCGTA

254 C L S P R M F Y Y K L Q E V Y N K A K H T S P P V

901 TCCCTGCTGGGTCAGCTGTTATGGAGGGAGTTCTACTACTGTGTAGCAGTAGACACACCCAACTTTGACAAAATG

279 S L L G Q L L W R E F Y Y C V A V D T P N F D K M

976 GAGGGAAACCCAGTGTGTAAACAAATTCCTTGGGACACCAATGAGAGTTATCTGAAGGCTTGGAAAGAGGGTCGT

304 E G N P V C K Q I P W D T N E S Y L K A W K E G R

1051 ACTGGATACCCCTTCATTGACGCAGTGATGACCCAGTTGCGACAGGAGGGGTGGATCCATCACCTGGCCCGACAT

329 T G Y P F I D A V M T Q L R Q E G W I H H L A R H

1126 TCTGTGGCCTGTTTCCTGACCAGGGGAGACCTGTGGATCAACTGGGAGGAAGGCATGAAGGTGTTTGAGGAGTAT

354 S V A C F L T R G D L W I N W E E G M K V F E E Y

1201 CTGTTAGATGCAGACTGGAGCCTGAATGCAGGGAACTGGATGTGGCTGTCTGCCTCGGCCTTCTTCCATCAATAC

379 L L D A D W S L N A G N W M W L S A S A F F H Q Y

1276 TTTAGAGTTTACAGTCCAATTGAGTTTGGAAAGAAGACAGATAAGGATGGGGACAACATCAGGAAATATGTTCCG

404 F R V Y S P I E F G K K T D K D G D N I R K Y V P

1351 CAGCTCAGTAAGTACCCAACAGCATACATTTATGAGCCATGGAAGGCACCGCTTAAAGTTCAAGAAAAGGCTGGT

429 Q L S K Y P T A Y I Y E P W K A P L K V Q E K A G

1426 TGTATTGTTGGGAAAGACTACCCCAAGCCTATAGTAGAACATGATAAAATCAGAAAGAAGAACATTGAAAGAATG

454 C I V G K D Y P K P I V E H D K I R K K N I E R M

1501 GCACAAGCTTACAATGCCAGTAAAACTGAGGGAGGAGCAGGTGCTAAAAGAAAGTCTGATTCATCTTCCTCACAG

479 A Q A Y N A S K T E G G A G A K R K S D S S S S Q

1576 AAGAAGAAAAAGGCAAAAGTGAAA**TAA**aaataccaagcaagttgcgattccaaggataatgaacatcttaagaat

504 K K K K A K V K *

1651 actgagaggctgatctgaaatctacctcacttcaataacatttgaattgttagtgaataccatgtccatttgtca

1726 ctgtttgaatatcactgtaattacataggagtagatgaactaccaataaaagtagtctatgcaaaaaaaaaaaaa

1801 aaggccataaggcgcctgatccttcgaggggggg

>CgClock

1 cgacaaaaatggcgacgcattatttgacatgttgtgatactgcaggaggagatacttagaaaggatactggaaaa

76 tggaaaactaacttactacaatggaaatatttttcacactgtattgat**ATG**AATTACGGGAAGCGAATCAAGCTA

1 M N Y G K R I K L

151 GTGCTCCCAAGCCGAAGTGAAAGTGAGTACTCGTTGGGGGATGAATATGATGATGATGGAAAAAGCAGTAAAAGC

10 V L P S R S E S E Y S L G D E Y D D D G K S S K S

226 TTTTTCTCTGATGACAGTAAGGGTGGATCCAACAAGGATGTTACTGACAGGGTCAATCGAAACTTGAGCGAGAAA

35 F F S D D S K G G S N K D V T D R V N R N L S E K

301 AAGCGAAGAGACCAATTCAACATGTTGGTGAATGAACTTTGCTCAATGGTGTCCACTTCCAGTAAAAAGATGGAC

60 K R R D Q F N M L V N E L C S M V S T S S K K M D

376 AAGTCTACCGTCCTAAAGTCAACCATAGCATACCTAAAAACATACCAAGAGACTGCAGTACAAGCTCAGGCCCAT

85 K S T V L K S T I A Y L K T Y Q E T A V Q A Q A H

451 GAAATCAAGGAGGATTGGAAACCTTCATTTTTATCCAATGATGAGTTTATGCATCTTATGTTAGAGGCATTGGAC

110 E I K E D W K P S F L S N D E F M H L M L E A L D

526 AGTTGCTTACTGGTTTTCACACAGCAAGGCAACATACTTTATGTTTCTGAGAGCATAACATCATTGTTAGGCCAC

135 S C L L V F T Q Q G N I L Y V S E S I T S L L G H

601 CTACCGGCCGACCTCACCAATCAATCTGTCTACAACTTTATGCACGAAAACGAGAAGCAGAACCTGTACAACATC

160 L P A D L T N Q S V Y N F M H E N E K Q N L Y N I

676 CTTTATCACTACAGCATGCTGTCGCCCGAGGACCGGGCAAAAGAGAAAGACCAGCTATGCTGCACCTGTCACTTC

185 L Y H Y S M L S P E D R A K E K D Q L C C T C H F

751 CGTCGAGGGGCAATATCCCCAAGCAGTAGCCCTCTGTTTGAAGTAGTCAGCCTGTCTGGGTTCCAACATTGGAGC

210 R R G A I S P S S S P L F E V V S L S G F Q H W S

826 AGGGATAAATTGAGTAGTGTGGAGGAAGAGAGCAGTCAATACAGCCTGAGCGGCCCCAAGGAGGACACCTGCTTC

235 R D K L S S V E E E S S Q Y S L S G P K E D T C F

901 TGTTGTACGGTCCGGCTACAGAACCCCCAGTTTATCCGGGAAATGTCCATGGTGGACGAGTCCAAAACAGAGTTT

260 C C T V R L Q N P Q F I R E M S M V D E S K T E F

976 ACCTCACGGCACAGTCTGGAATGGAAGTTTCTATTTCTAGACCACAGGGCCTCTCCTATTATTGGGTACCTACCA

285 T S R H S L E W K F L F L D H R A S P I I G Y L P

1051 TTTGAGGTCCTAGGGACCTCTGGATATGAGTATTACCATCCAGATGACCTGGATCAGATAGCCAAGTCACATGAG

310 F E V L G T S G Y E Y Y H P D D L D Q I A K S H E

1126 CAATTGATGCAGACAGGGGAGGGAACTTCGTCATACTACCGATTCCTGACCAAGGGACAGCAGTGGATCTGGATC

335 Q L M Q T G E G T S S Y Y R F L T K G Q Q W I W I

1201 AAGACTCGCTACTACATAACGTACCATCAGTGGAACTCTAAACCCGAGTTTATTGTGTGTACCAATGTAGTCGTT

360 K T R Y Y I T Y H Q W N S K P E F I V C T N V V V

1276 AGCTATGCCGATGTTAGAGAACAAGTTTACAAGGATTTAGGGATTGACTCTAAATCGGAAAACTCTGAACCTTTC

385 S Y A D V R E Q V Y K D L G I D S K S E N S E P F

1351 CTCCGAATTCAGCGAAGTCCCTCTGTCTGTTCTGTTACCTCAGGAGGGAGGTCGCGCTCCAACTGGTCATCTTCA

410 L R I Q R S P S V C S V T S G G R S R S N W S S S

1426 CACTCCATTGACTCGGCCAGTACAGATCAGCACGTCAGTGGAAACCACATGTCTGATAAAGAGAGTGTGGAGGAA

435 H S I D S A S T D Q H V S G N H M S D K E S V E E

1501 CTTCCGTCGCATATAACGAAAGATCTCCCGCACACAATGACTCAACACAAACACTTACAGTTACTTCTCCAGCAG

460 L P S H I T K D L P H T M T Q H K H L Q L L L Q Q

1576 CGCTACCTTAACCAAGCCACCAACAGTGAGCCCAAGCCTCAGAGATACACGGAGCCCGCGGCCAATAGCAACAAT

485 R Y L N Q A T N S E P K P Q R Y T E P A A N S N N

1651 TTACTACAGGCCGGCCCAGACAGGATGGGAATGATGTCCAACAGAGGTGGAGCTGACAATGTCTCATCGACGTCT

510 L L Q A G P D R M G M M S N R G G A D N V S S T S

1726 CACATTCCGGTGATACATCCTGGTCGACAACTTCCTGTCATTGATGGACCAAAATCTCCGGTACCACAATTATTC

535 H I P V I H P G R Q L P V I D G P K S P V P Q L F

1801 CTGACTCCAATACAGAAGCGGCTCCATGAACAGCTGAGAGAGAAGTCCCAAAAACTACAACAGGCTATTCTAAAG

560 L T P I Q K R L H E Q L R E K S Q K L Q Q A I L K

1876 CAACAGGAGGAACTCCAACAGATCACACAGCAACTGGCTATGGCTCAGCAAGGAATGCTCTCCCTGCCAGATACA

585 Q Q E E L Q Q I T Q Q L A M A Q Q G M L S L P D T

1951 TCTATCCCTAACCCAGGTGCGCCTGTTCTTATGAATCCACCAATCGGAGCCACTGTGATGACCAGTGCCCCACTT

610 S I P N P G A P V L M N P P I G A T V M T S A P L

2026 AACGTGTCGGCGGGATTTCCCATGCAGACGTCGGTAACACCATCCCTACCCCCAGAGCAACAACAGTTCATCCCA

635 N V S A G F P M Q T S V T P S L P P E Q Q Q F I P

2101 TTCCAGTTATTGCCTCATGACACAGAATCAGTGTTCTCTTACACCTCAGAC**TGA**ggccttgtccattctcaggtc

660 F Q L L P H D T E S V F S Y T S D *

2176 acttagaaactcatgttacaagattgtttgtattggagaagagagtgccatttcactttgtgaatgtgtagtttg

2251 tgtagtgtttcacagacatttttctgccaaggcatgaaaatataacgagctgctacatgtgtgttttgtctttgt

2326 tgttgggttatcattagacatatactttaaaccaacttttatgcgcttgcaaaatatttttgcaatgttcgcaag

2401 agctttatcgtttgcaaatatttctcgccccgattcagtctttaaatgcctatggtatattaccgtattttccac

2476 attgtctacatcttgtttgcaaaaatagtagctgccattatatcttcagtaaatcacaaaataaatttgtcgcaa

2551 ataaaaattggtttacagtatatgtaaatgttctcatcatgatgcaggggaagacagaaaaattgagtcattttc

2626 ttcatagagccagtgttaattttgaaactaacatgtttgtagaattcattttctactgaagaaagaatgttagct

2701 gttctcttcattatatttatagctgtttagggaaaatgcaatttcattgcaataatccaagattaaaaagataag

2776 cgatgttgcccacatattaaatcatgtcatttattgtcatgtctggttatgcaatagaataagttgataatcctt

2851 ataaaatggtgtgaatgatgtaagcaagtaggtgcctatggttcaaataacaaataaggttagtacattaactca

2926 tcaaccagaatttattatgttgaaaattgatgctatcataatgcttttgtatacatgatgttttgatataggata

3001 tatttacttcaatgtacatgtgtatgggtaaacatttgtttgatataagaattattttattcaactttatatgag

3076 tgcattgaaaaactaaatacctgtatatgttctctcgttaatattttttaatatttgcaactcattttttttttt

3151 ttacatcaataactctatcgtcattgtataggttacaacctgttatgttctctctgacatgatttgaacttctgt

3226 atccatgactgtaactttatcaagtctgtaatttctaccaaagattgcaaccagtgtctccgacctctacgtcac

3301 tggctataacctctctataaaaatttacttgtttatcaatgaccttgacctctgtgtcaataactttgacctcta

3376 tgtcagtgacctcaaccactgtcaatgactttgacctctgtgtcagtgaccttgacctctttgtcagtgaaatca

3451 agctctctgtcaatgactttattactgtcaatgagtgtgattatttttcatggactgaggtttctggtttcagtt

3526 taatttttttgtcactcaaaaaaaaataattatcaacttcaacttctttagccttgtacacagttttagcattgt

3601 acaaagtctttctgcagcatttctctccatctctctacaagtactggctttaaatctttgtttatgacactgggt

3676 taaatattgcacacttacggtatcaatgtttgggttgctaatcattttaactttgagtttgat

>CgCry2

1 tttaattcggcatgctaatatggcgcagatattgacggtatgtttccaaaaaactgtgtcgtgtttggtgatgta

76 gagggggtatagagtgtcacagttcgaaggggaaaacagacttaaatt**ATG**TCAAAAAGTAGTAAAAGAAAACAC

1 M S K S S K R K H

151 GTTGTGCATTGGTTTCGGAAAGGACTGCGCTTACACGATAACCCATCTCTACGCGAGGCGTTGAAGGGTAGTTCG

10 V V H W F R K G L R L H D N P S L R E A L K G S S

226 TCTTATCGGTGTGTTTACATCCTCGATCCTTGGTTTGCTGGGTCATCGCAGGTTGGGATCAACAAATGGAGGTTT

35 S Y R C V Y I L D P W F A G S S Q V G I N K W R F

301 TTACTGCAATGTTTGGAGGACCTTGACACATCTCTGCGGAAATTGAATTCCAGATTATTTGTTCTACGGGGCCAG

60 L L Q C L E D L D T S L R K L N S R L F V L R G Q

376 CCCACAGACTTATTCCCAAAGATATTTAAGGAATGGAACATCACAACCCTGTCATTCGAGGAAGATCCGGAGCCG

85 P T D L F P K I F K E W N I T T L S F E E D P E P

451 TTTGGCAAGGAGCGGGATGGAGCCATCCAGATGTTGGCGAAGGAGGCGGGGGTGGAGGTCATCGTCAAGACTTCC

110 F G K E R D G A I Q M L A K E A G V E V I V K T S

526 CACACGCTCTACGACCTTCAAAAAATAATCGCTATGAATGGAGGCTCCCCTCCCCTGACTTACAAACGCTTTCAG

135 H T L Y D L Q K I I A M N G G S P P L T Y K R F Q

601 TCAGTCCTCGCCAAAATGGAGGCTCCTAGCGAACCAGAGGAAACCATCAACAGCGGATTCCTGGTGAAAACGAAA

160 S V L A K M E A P S E P E E T I N S G F L V K T K

676 ACACCGATCGCAGAGGACCACGACGACAAATACGGGGTACCCACGTTAGAGGAATTAGGTTTTGATACTGAAGGT

185 T P I A E D H D D K Y G V P T L E E L G F D T E G

751 TTGGGTCCGGCAGTGTTTCATGGCGGGGAGGCTGAGGCTTTAACCAGACTAGAAAGACACCTGGAACGAAAGGCC

210 L G P A V F H G G E A E A L T R L E R H L E R K A

826 TGGGTAGCCAGTTTTGAGCGACCCAAGATGTCCCAGCAGTCCTTGTTTCCCAGTCAAAATGTCCTCAGTCCCTAC

235 W V A S F E R P K M S Q Q S L F P S Q N V L S P Y

901 CTTAGATTCGGTTGTCTGTCTGCTCGTCTGTTCTACTGGAAGCTGAGAGAATTATACAGAAAGGTAAAAAAGCGA

260 L R F G C L S A R L F Y W K L R E L Y R K V K K R

976 AAAGACCCTCCATTGTCCCTGCATGGTCAGCTGTTATGGAGGGAATTCTTCTACACGGTGGCCACAAACAACCCA

285 K D P P L S L H G Q L L W R E F F Y T V A T N N P

1051 AATTTTGACCGAATGAAAGATAACCCTCTCTGTGTGCAGATACCTTGGGACAAGAACCCTGAAGCTTTGGCGAAA

310 N F D R M K D N P L C V Q I P W D K N P E A L A K

1126 TGGGCAGAGGGTAAGACTGGGTTCCCGTGGATTGATGCTATCATGATGCAGCTGAGACAGACCGGATGGATTCAT

335 W A E G K T G F P W I D A I M M Q L R Q T G W I H

1201 AACTTGGCCCGTCACTCAGTGGCCTGCTTTCTGACCCGCGGTGACCTCTGGATCTCATGGGAAGAGGGAATGAAG

360 N L A R H S V A C F L T R G D L W I S W E E G M K

1276 GTGTTTGAGGAGCTATTACTGGACGCGGACTGGAGTGTCAATGCAGGCATGTGGATGTGGCTGTCCTGTAGCTCC

385 V F E E L L L D A D W S V N A G M W M W L S C S S

1351 TTCTTCCAGCAGTTCTTCCACTGCTACTGTCCCGTCGGCTTTGGTAAAAGGGCCGACCCCACGGGCGACTTTATC

410 F F Q Q F F H C Y C P V G F G K R A D P T G D F I

1426 AGAACATATTTGCCAGTGCTTAAAGGCTACCCAGCGAAGTACATCTATGAGCCATGGACAGCCCCAGAAAGTGTC

435 R T Y L P V L K G Y P A K Y I Y E P W T A P E S V

1501 CAGCGTGCGGCCAAGTGTATCATCGGCGAAGACTGCCCAGTGCCCATGGTGAACCATGCGGAGGTCAGCAAACTG

460 Q R A A K C I I G E D C P V P M V N H A E V S K L

1576 AACACGGGGCGCATGAAGCAGGTGTACCAACAGCTGGCTGTGTACGCCTCGATAGCATCTGTTCCTAAGCAAATC

485 N T G R M K Q V Y Q Q L A V Y A S I A S V P K Q I

1651 CACTCAGAAGAACCTTACAGCAAGCACGAAAAAGCAATGCATTCTGGGAACCATCCATCACGGGTAGCCATGTTG

510 H S E E P Y S K H E K A M H S G N H P S R V A M L

1726 GAAAACACTGACCGTGGAAACCACAGCCAGATGTCAGCA**TAG**gaagaggaagggcaacagcaacagttcctggca

535 E N T D R G N H S Q M S A *

1801 gcatagcaagaggagccggtgattggagacttcgaacattgaacgcattgatgtgttgtagaaacttctactttt

1876 ctaaggagtccacaaacaaaaagttgatatatgtgttgtagaaacttctactgtcgttaggagcccaccaaaaaa

1951 aggggatgagaattattgatgtgtcacaaacttcaactgttgagggagaactgttgagacatgctttcagaggga

2026 gacttctcaaaatgacactactgtgcaagttactgaatggtaaatatttcttcagccttggtgcaaatgttattg

2101 atcacgtgggatatagtgtcatggactctccttttgagagagtggaaattgcgttatcggatcatgatgatgtat

2176 cgcaaatgtagattgtgctggaatttgaggtggatcggacattttataatgaaaggttaactccatcattatcag

2251 tgagttgagtggaataattcttcgaattgacatgtttccaggaaattttgaagatt

>CgBmal

1 tcaaggttgcccgtgatcatgtcagacttggaacttgtgtgtggataaaaatggaattgtacggcttgtgattat

76 gaacgaaacctagacttcaacctctttgacaatctgtttctaccaccagaccaaggcagcctg**ATG**GAACCCTCC

1 M E P S

151 CTGCCTGGACTGGACAAGATTCCAGGGGGCATAGGTCCATACCTGAATCACCACAGGAAGCGGGGGTCCGTAGAT

5 L P G L D K I P G G I G P Y L N H H R K R G S V D

226 TGGGAGACCACCAGCCCAACTATGGACACAAACGAGGACGATTCATTCATGGATTTCGATCACAGAAAATATAAC

30 W E T T S P T M D T N E D D S F M D F D H R K Y N

301 TCCAAGGCAAAAAATAGTATGATGGATCCCCAGTCAGGCAATGACAGGCAGAACCACAGCGAGATTGAGAAGAGA

55 S K A K N S M M D P Q S G N D R Q N H S E I E K R

376 AGGAGGGATAAAATGAATGCGTACATCACGGAGTTATCCTCCATGCTGCCCGTGTGTAATGCTATGAATCGAAAG

80 R R D K M N A Y I T E L S S M L P V C N A M N R K

451 CTGGACAAACTAACGGTCCTTCGAATGGCAGTGCAGCATTTAAAGTCTTTAAGAGAGGGTGCTGCTATGTCCATT

105 L D K L T V L R M A V Q H L K S L R E G A A M S I

526 CCAGAGGCCAGACCCTCCTTCCTGTCTGATGATGATCTCAAGCATCTCATTCTAGAGGCAGCCGAGGGTTTCCTG

130 P E A R P S F L S D D D L K H L I L E A A E G F L

601 TTTGTTGTAAGCTGTGACAGAGCCAGGATTCTCTATGTCTCCGAGTCAGTACGCAACATTCTCAACTATACCAGG

155 F V V S C D R A R I L Y V S E S V R N I L N Y T R

676 CTTGATTTGATTGGTCAGAGCCTGCTGGATTACCTCCACCCCCACGACATCAACAAAGTCAAAGAACAGCTGTCT

180 L D L I G Q S L L D Y L H P H D I N K V K E Q L S

751 GCCTCCGATGTTTATCCAAGGGAGCGGCTAATTGATGCTAGAACAATGATGCCGGTAAAGACGGAGATGATACGA

205 A S D V Y P R E R L I D A R T M M P V K T E M I R

826 CGACCTACTTACCTCTGTTCTGGGGCCAGGCGCTCCTTCTTCTGTCGGATGAAGTCCGGCAGTTCCATGTCATAC

230 R P T Y L C S G A R R S F F C R M K S G S S M S Y

901 CTCGGACTCAAATCCGAGAAAGAGATGGACCTGGAACTCTGTAGTCGGAAGAAAAAATCAGACAGGAAGTCCTTC

255 L G L K S E K E M D L E L C S R K K K S D R K S F

976 ACTGTGATACACTGTACAGGGTACCTCAAATCCTGGCCCTCCTCCAGCCTGGACATGAAGGAGCAGGATGACTCG

280 T V I H C T G Y L K S W P S S S L D M K E Q D D S

1051 GAGGATAACTGTGATTTAAGTTGTTTAGTGGCAGTAGGACAAATCAAAACGTCGTGTGACAAAAAAATCATAGAC

305 E D N C D L S C L V A V G Q I K T S C D K K I I D

1126 AGAGACAGCAATATCAATGTCCGTCCAATAGAATACGTGTCGCGGATGAGTATCGATGGCAAGTTTACCTTCGTT

330 R D S N I N V R P I E Y V S R M S I D G K F T F V

1201 GATCAGGGAGCGACAATTCTGCTAGGATATCTACCTCAAGAGCTGCTAGGGACATCTGTGTATGAATTCTACCAT

355 D Q G A T I L L G Y L P Q E L L G T S V Y E F Y H

1276 CAAGAGGACATAGCCAGCATGTCGGACATACATCGAAAAGTTTTAAAATCCAAAGAAAAGGTGAAAACAAATGTC

380 Q E D I A S M S D I H R K V L K S K E K V K T N V

1351 TACAGATTCAAGATAAAGGACGGTTCCTTTATTCACTTGAGGTCGGAATGCTTTAGTTTCCGGAATCCATGGACC

405 Y R F K I K D G S F I H L R S E C F S F R N P W T

1426 AAAGAAGTGGAGTACATCGTATCAACCAACACTTTTGTCCCGGAACAAGAGGTCACAAGTTCAGGTCAGAGTGTA

430 K E V E Y I V S T N T F V P E Q E V T S S G Q S V

1501 GATGCCGGGTCAGAGGTCATGGACAGCAGCAATAACTGGGAGTTTAAAGATGATAACCAGTCTACCTCAGGTGAG

455 D A G S E V M D S S N N W E F K D D N Q S T S G E

1576 AAGAAAAAGTCTGAGGCAGGAGCTGTTGCCATGGGGACCAAGCTTGGTGCTGGCAGGATAGGGCGACAGATAGCT

480 K K K S E A G A V A M G T K L G A G R I G R Q I A

1651 GAGGAAATGATAGAAATGCAGAGGGGCTTTAACACAACACTGGCGGACAGTACAAACTCTGTCCCTGTTGCTAGC

505 E E M I E M Q R G F N T T L A D S T N S V P V A S

1726 CCAGCAGCCATTCCGGGAAAACATTTTGGATTAACAAATGGACTTGCTGTCAAAGTTCCAGTTCCTTTATCTGAA

530 P A A I P G K H F G L T N G L A V K V P V P L S E

1801 CCCCCAGTTGCAACAGCAACAGAGGTTGCTGTGTCCAGTGAGGTTGCAGCAACTTTAGCGAGCCAGGTGGCTGTG

555 P P V A T A T E V A V S S E V A A T L A S Q V A V

1876 ACGAGAAAGAACGGCTTGACTGTTCCCACTCAGTCTACCTCCACAGCGGGATCAGTAGCCAGTACTTCAGCCGAG

580 T R K N G L T V P T Q S T S T A G S V A S T S A E

1951 ACTAACCTGATCGAGGGTGTCATCGCCGAGCAAGAATCAACGGAGAACGAGACGGGACAGAGTAGCGAGCACGGC

605 T N L I E G V I A E Q E S T E N E T G Q S S E H G

2026 AATGACGAAGCGGCCATGTCGATCATCATGAGTCTACTCGAAGCTGACGCAGGTCTCGGAGGACCTATTGACTTT

630 N D E A A M S I I M S L L E A D A G L G G P I D F

2101 AACGATCTCCCCTGGCCCCTG**TAA**ggacacagtgagataggaggacgcagtgtccatcttcacagatgtacagag

655 N D L P W P L *

2176 atagagggagggcggtcaggcttcaccctgccggtgttgctggcatcatatgactgtgagatcctgagggaggcc

2251 ggacctacaaggctcaaagtcttggtccaggaagtggcaatgtgttcagggaagaggtggattaaaccgcgccaa

2326 gggtttactttgcaatggaagtgttacacagaggttttttatcgtgtttagaattgttgttgagcagcaaactac

2401 aagtgagagagaagataaagtgtgaagtgctagagagagtgtgtgattggtttaaatgtggttgcgagtatgttg

2476 gacagcattagatttttttgtgaatgcatgagcaatttctgtgatgtgtagatgcaatgtaccggtatattgtta

2551 tattgtccttgtgcctgttatatatagatataagaagtgctagtaacaaattctcactccgtttaaacagtgcct

2626 caattatattacagtggtccattaaggtatctggctcacctgtcatacaagcggccttcagctttatcaaaacat

2701 caagtagactaaaatcaggaacatattccacaaatatcatgttattatttatttcatttcattttcatttaaatg

2776 tacatcactcaagcattactatctctttcttacatacaaaaagttttgtttttcccaaaaagccagattttttat

2851 aagtaatcagtgtttataccttaagtaacaacttttcgaaatctatctgatgtagtcccaatatgtcacttcaca

2926 gtcactgaattcatcccctgtccctgtaaatattaatacctcatatatgtgaacagaaattttcaatttgtagaa

3001 taatctttgtggaaaagaaatggttctcgactgttcatctaaatttttatatgtccc

>CgPeriod

1 atttcaatacagtgatttctgtaactgtttatttattcaacctttagtacgtacaaattcactggatttattcaa

76 tgtgggagttgtcgctctgtgtcaatttcaacaacatgtttgttgttaaatggaagattgattaat**ATG**GAGGAA

1 M E E

151 TGTTTTGTCAGTGATTCTACATATGGATCTCTAAGAAGTGGAATGCAAGACAGTAGCAGCAGTTTCAGCATGTCT

4 C F V S D S T Y G S L R S G M Q D S S S S F S M S

226 CTCAGCGGTAGTGATACTTTTGAGGACCAGCCCTCCACCAGTGGCTGCAGCAGTGACATGACCCACAAAGAAAAG

29 L S G S D T F E D Q P S T S G C S S D M T H K E K

301 CGTAAAGCTCGAGTGAAACAATATCTGAGGCAGCTTAAGGCCATGGTGCCTCCATCTTCAAGAAAAAAGGGGAAA

54 R K A R V K Q Y L R Q L K A M V P P S S R K K G K

376 ATGGGCACCCTTAGTGCCTTACAGCATGTAATAGGAAGTCTCCAGAAAATCCAAGAAGAAAAAGATAAAAGTCAA

79 M G T L S A L Q H V I G S L Q K I Q E E K D K S Q

451 TTAGCAAGTGGTGATGCCCTTGTTGAAGAACTAGACAATTCCCTCTTTGATAAAGAATACCAACTCGACTCACAA

104 L A S G D A L V E E L D N S L F D K E Y Q L D S Q

526 GTCCTAAAAACAGAGGAAACTGTTCATGTTGTGCTAACCATGAATGAATTTGCAGTCCTCAAAGTGTCGGAAAAT

129 V L K T E E T V H V V L T M N E F A V L K V S E N

601 GTCACTCAAATCCTAGGATATCCAACAGACAGCTGGATCAACAGAAGTCTCAGTCATTTTGTTCACAAAAAAGAC

154 V T Q I L G Y P T D S W I N R S L S H F V H K K D

676 ATAGTTACCATCCATAACAGTCTCAATGTGGAAGAACTCGTACAAGTGACGGATTATGATGATTCTCCAGTTGAG

179 I V T I H N S L N V E E L V Q V T D Y D D S P V E

751 AGCAAACCCGAAGTTAAAAAAGTCAGCAGGCAAATAAGGAAAAAGTTCTTCTTCAGAATAAGAAACTACAAAGGG

204 S K P E V K K V S R Q I R K K F F F R I R N Y K G

826 TTACAGCATAGTGGCTTCAGCTTGATAAAACCTGACCGTTTCACAACTGTCCAAGCCACCATGTCCATTGGTTAT

229 L Q H S G F S L I K P D R F T T V Q A T M S I G Y

901 TATCCAGAGAGAAACAAAGCTGACTCCCCGTCATCCTTCTCCTCCTCTGAGGACTGTGTACAGAAGGGGAGGAAA

254 Y P E R N K A D S P S S F S S S E D C V Q K G R K

976 TGTATCTTCCTAGACTGTACTCCCCTACGTCCACTCTATAATGTTGAAAATGTACAGTTAGATAAGCAGACTTTC

279 C I F L D C T P L R P L Y N V E N V Q L D K Q T F

1051 CATATGAGGCATACCATCTATTGCAGTTATAGCTACATGCATCCAAATGCCATTCCCCTGTTAGGATACTTACCA

304 H M R H T I Y C S Y S Y M H P N A I P L L G Y L P

1126 CAGGACTTAAATGGGATGTCAATCTTTGACTTTTATCACAAGGACGATCTGGAGACATTGTGCAACATTTACAAA

329 Q D L N G M S I F D F Y H K D D L E T L C N I Y K

1201 CGAATTGTAGTCTCCAAAGGAACGACGTTCAAGAGTAAGCCAATCCGACTGAGGACTAGGAATGGTGACTGGCTG

354 R I V V S K G T T F K S K P I R L R T R N G D W L

1276 ACAGTGGAGACGGAGTGGTCAAGCTTTGCCAACCCCTGGTCACACAGACTCGAGTTCATCATTGGTCAGCATAGA

379 T V E T E W S S F A N P W S H R L E F I I G Q H R

1351 GTGATCAAACCACCAACTGATTGCAATGTCTTCTGTGAGGCAGAGCAACTTACTATGATACCAATGCTCAATGAC

404 V I K P P T D C N V F C E A E Q L T M I P M L N D

1426 CACCATCAGAAGTTACAGCAGAAAATCAGACAGATGTTGTTAGAGCCCGTGAGTGAGGAAAAGACCGTTGTCAAG

429 H H Q K L Q Q K I R Q M L L E P V S E E K T V V K

1501 CATGAACCAACGACAGATTCTTCTGATGACCTGAGGACTTCACAAGAAACAGAAAAAGTTCAGAAAAAGAGCAAA

454 H E P T T D S S D D L R T S Q E T E K V Q K K S K

1576 CAGCAAACAGAAAAATATGTGGACAGAAAAGCTGGAAAATGCTCGGAGTCCCTGCAGTCCACAGAGTCACTAGGA

479 Q Q T E K Y V D R K A G K C S E S L Q S T E S L G

1651 AGAAGTTCACTCCAAACTTTTCCAGAGCATGAAACTTCTATGGCATATGAGCAGTTAAATTATGCCAATAGTATT

504 R S S L Q T F P E H E T S M A Y E Q L N Y A N S I

1726 AAAAGATACCTAATGAGCCAACAAAAAACGTATTCATCATCTTCAGAGAAGAAAACAACATCTGAGGAGGAAACT

529 K R Y L M S Q Q K T Y S S S S E K K T T S E E E T

1801 GATACACCTTGCACCATCTCCACAACTACAGCAGAAGCATCAGACTCTGAGTTTGAAGTTGATATCTCCGTTCCA

554 D T P C T I S T T T A E A S D S E F E V D I S V P

1876 AAACCACCAAGCTTTGGGAGCAGTACAAAGGTACTACTGTCAGAGCAAGAACAGAGGGAGGACATTGCTGGTAGT

579 K P P S F G S S T K V L L S E Q E Q R E D I A G S

1951 CCCGCACACCAAATAGAGGATAACACAGAGGAAATATCCACCTCCCCTGGGGCTGTTGCACCCACCTCCATGTTA

604 P A H Q I E D N T E E I S T S P G A V A P T S M L

2026 CCCCCACCCTGTGTGGAGAATATGGACACGATCCGCCTGTTAACCCTGACGCAGGACGCATTGTTACGACACACC

629 P P P C V E N M D T I R L L T L T Q D A L L R H T

2101 AAACAACAGGAGGACCTGTTTGTAGCTCATGCCAAGCAGGATAGAACTCCTATCATCCTCAAATCAAAGGAAGGG

654 K Q Q E D L F V A H A K Q D R T P I I L K S K E G

2176 GGCATGCTTCAAGAGAGGAAGAGAAGTCATTCCCCCGACCATGAAAAGGGCTTCTACAGGCCGACCAAGGCCTCG

679 G M L Q E R K R S H S P D H E K G F Y R P T K A S

2251 AGAAATGACAGCAATATTTTGATCCCACCATTTCCCTTGCCAAATATAGGGTACCCGGTTCAGTACAGGCAAGGT

704 R N D S N I L I P P F P L P N I G Y P V Q Y R Q G

2326 AATGCCCCTAGAGGTGGGAATGTGAATGGAGGTCAACAGATTGTGTCTCAGTCTAGGCTTCCTGTTTCTTCTACC

729 N A P R G G N V N G G Q Q I V S Q S R L P V S S T

2401 AAACTGTCACAGAGTAATGTAGAAAGTAGAGCGACTCCAATACAGAGCAATGTGATTTGGCCATACTACCCTCAA

754 K L S Q S N V E S R A T P I Q S N V I W P Y Y P Q

2476 ACGGCATCAGGGGCCTCATTCTATCCACAAGTGATGGGAGGATTTTATCATGACCCCAATGGTATCCCAGCCACC

779 T A S G A S F Y P Q V M G G F Y H D P N G I P A T

2551 TTGAGTCTCAATGTGACAAGTACTATACCCCCAACAGCCAATACGACTGGTACAACAATGAGTGGCGCTTCTAGA

804 L S L N V T S T I P P T A N T T G T T M S G A S R

2626 CACAATCTAAAGGTACTGCAGGGTCGTTGTCAGGGGCCGTTCCAGCTGCCCACCATCAGCACCACATTTTCTTCC

829 H N L K V L Q G R C Q G P F Q L P T I S T T F S S

2701 TCCTCGGACATGTCCATCTCCCACACAGACAGTGGCTCCTCCTACCTTTATCTGCTAGACTCGGACGACCAGAAT

854 S S D M S I S H T D S G S S Y L Y L L D S D D Q N

2776 AGCTCAGGACAAGAGTTGGAGACCAAGAGTAATAAAGTTACCAGAAGTAATAAACGGCAAACAGAGCCACCATGG

879 S S G Q E L E T K S N K V T R S N K R Q T E P P W

2851 CTGGAGACCATATGTTGGACAAAGAAACTTGCCATGAATTACCAGCTTCCAAAGAGGAAGAAGAACAGGGTGCTG

904 L E T I C W T K K L A M N Y Q L P K R K K N R V L

2926 AAGTCTGACGAAGCTTTCATTGCCAAATCCAAGCCATCGAATACTCTCCTTCAACAGATGATGGATTTACAGAGT

929 K S D E A F I A K S K P S N T L L Q Q M M D L Q S

3001 GAGATAGACCTGAATCAGGGGGCACCTGCTGTGGATGAAGAGAATGACTATCTTTTCTATTTGGATGATGAGGAG

954 E I D L N Q G A P A V D E E N D Y L F Y L D D E E

3076 GAGGATCAAGCATCAGATTGTGATAGCAGGTTTATTGCCCTGCAAGACATTCACGAGGCTCTCTCTCAGTGTGAG

979 E D Q A S D C D S R F I A L Q D I H E A L S Q C E

3151 AATCACAGTTCTGGCATAGGGTGCTACGCCCAGCAGGCACAGCCCCACTGTTCACCGTCCGATGACAGAAATCCC

1004 N H S S G I G C Y A Q Q A Q P H C S P S D D R N P

3226 AGTAGCACAAAATCCTCAGAAAAACTGGAGGATTCTAGTGGAGATGGTATAGATATTTCACTGGGCCCTGAAGAG

1029 S S T K S S E K L E D S S G D G I D I S L G P E E

3301 GAGCCTCGATGTGAAGGTTCTCAAGAGCAGGAGAATAGGATGGACTGTCGGACATCGATAGAAAATCAACCCTCT

1054 E P R C E G S Q E Q E N R M D C R T S I E N Q P S

3376 CTGGAAACTCAATCTCAGTTAGAGACACAGGAAGCCATGGATAATCAAGGACCTTTAGACACTCTATCCATGGAC

1079 L E T Q S Q L E T Q E A M D N Q G P L D T L S M D

3451 ATTGAATCCAATTGTTCTAAATCATCGAGTGACTTGACGCCTAGTGATGAGAGGAGCAGTGGTGAGGCTGGAAGT

1104 I E S N C S K S S S D L T P S D E R S S G E A G S

3526 TCTCTGAAGGAGTCGGATGCCATGTCCTCAAAGGATTCTGTTGATGAGAGCAAGGACTCTTCTGAATCTGATGTG

1129 S L K E S D A M S S K D S V D E S K D S S E S D V

3601 GATGTGAGCAATAGCACAAAGAAGGACAACTATCATTTGTATTTTGTCATTCCCCCTATTTCCTTTGTGGATCAT

1154 D V S N S T K K D N Y H L Y F V I P P I S F V D H

3676 GGCAAGACTCCATTTTGGTGTAGGAATTGTGAAATGACTCCTGTTGTAGAAATGGAGTACACAGTCAAGTCAAAA

1179 G K T P F W C R N C E M T P V V E M E Y T V K S K

3751 GACTGTAAAAGACATCTAGATGATGACATGCGAAGTTTGAAGGATGTTGTGCAACCAGATCTTGTGAAAACACAA

1204 D C K R H L D D D M R S L K D V V Q P D L V K T Q

3826 ATGGGCTACATGCTGGAAGAGTTGAACTTATCTAAGAAATTGGAAGAAAAGATTGACAGTGGAACTAAAGAAACT

1229 M G Y M L E E L N L S K K L E E K I D S G T K E T

3901 GAAGGAAATCGTGGTTTATTGAAGAAATCAGCATCACATTCTAAACCTAAATCAGAACACTTTCTGACTGAGGAT

1254 E G N R G L L K K S A S H S K P K S E H F L T E D

3976 GTGTTTGATGGCATGTTTGTGACTATGTTGGGTGAAGATATTGTCCCGTTTGCAAGAGAAGAATCACCAGATGTT

1279 V F D G M F V T M L G E D I V P F A R E E S P D V

4051 GAGGATGTTTCTCACCGACTAGAAAAAGTGGAT**TAA**agtgttgaattacatgtattgaacctgattggatgtatt

1304 E D V S H R L E K V D *

4126 cttcctttgattatcacttttatatgttgttcgtgaaacgaaaattcattggcttatcagcaattactattgagg

4201 tgaccttgatttatgagtctactgttgcaaaatgtattttgaacgtatgagattttcccattagtgctgtaaaga

4276 ttttattaacagaattttctttggttttcttctgaaagtttatcactt

>CgTim

1 aaaatagtttagaaataattaatatataaatttcatgcataaataaacatgaataatttttacttataaaatatg

76 cataaatatcacacatatatattcacatttgtgcaaaaggaaagaaagttttaacacattttgatgtttattgga

151 aattcaccctcttggggccccatacaaacaccgatacaccgggctcaacaaaactgcctttcttgcaatataccg

226 gtatcagaatgaacagtaatgaatgaaacatttttttgtttacatatctaaaacttgtaaccatcaatgtattaa

301 aatcagacatatattttacatggaaaagggtgtagagttctgtatgtaaaaacttaccagtccagtaagagtttt

376 gccacttcagcattagtctcacaggctagtttgcttctcacttcattccaccgatcaatttgatcccctatcgat

451 atcctgcacttactatctgctgcatttctttcctttttgcgacgttttctatcagaatcagtcaatttcactttc

526 ggaccacgtttacccgacattgtttacaactttcgctcatgcgcaattaaggcattccgaagtagtgacctctga

601 ggtcactgcatatcgagataacggctgggaattagtggctggcagccagtaattcagattttgggttttaactgg

676 cgttttagcggttgtaggagcttacgatcctgaaaatcatggattatacaaacaaaaggataatttcatataatt

751 ttgtttgttttctacatatttattttttgtaccgtcagttatagctcatatggctttaatgataaagcctctttc

826 aacgtgaatgatcggctcgtgttcacttcaccgtcatgatcaatacacgtgacacgtgaccaaacatggaggata

901 ggaaaacaaacttgtgcacctcgagctcaaccgctgcttaaaacgatggaagaatcagcaacagatacctagaac

976 aaagttcatcaacttggaaacagttatgaatatgtgatttaaggaacctccataacttaagaaactacaagatta

1051 gcagagatttgaacgacaattgaagcagccacc**ATG**GAGTGGCATATAATGAATGTTGGGGGGGTGAGCAGTCTG

1 M E W H I M N V G G V S S L

1126 GGCAGTAACCTGGGCTGTCTGGAGGAATCTCAGTACATACCCTCTGACGAATGTAAAGATGTTCTTTTGGAAATC

15 G S N L G C L E E S Q Y I P S D E C K D V L L E I

1201 ATGCAACAACTAAAAGATGAGGACCCTAGGTTACGAAATGTTCGGCGGCAAATTTTCTACAACCATCTCGTGGAA

40 M Q Q L K D E D P R L R N V R R Q I F Y N H L V E

1276 AAGGATCTTTGTGTAATGATCAAGTTCTTGAGAGACGACCCAGAAATATTTGGTCTTGTTGTAAGAATACTGGCA

65 K D L C V M I K F L R D D P E I F G L V V R I L A

1351 AATTTGACACTTCCTGTGGAAAGTTTAATTGAGTCGACTGGATATTCTATGGCAGAGGCAAGTCAGACTGGAATG

90 N L T L P V E S L I E S T G Y S M A E A S Q T G M

1426 TCATGGCAACAGGGAGTGGAGAGCTCCCTCCACCACTATAGGACTGTGTTCACTGATAAGTCAGTTTTGCTTAGT

115 S W Q Q G V E S S L H H Y R T V F T D K S V L L S

1501 CTGTTGGAGGAAATCCACACTCTTGTGGAAGAGAGTGAAGGATACCCACTGCCAGAAGAGAGTTGTGATGTCATC

140 L L E E I H T L V E E S E G Y P L P E E S C D V I

1576 AATCAGATTCTGCTGTTGCTGCGGAATCTCCTACACTTCCCAAGCAGTTCTGAGGACAACAGCTCAAGGGAGCAT

165 N Q I L L L L R N L L H F P S S S E D N S S R E H

1651 GAAATGTTTCTCAGAATCCTTTTTGAGTGCGATTTCACAGGGAGATTTATTAAGCTTCTTCAGCTAATACAAAGG

190 E M F L R I L F E C D F T G R F I K L L Q L I Q R

1726 GAATACTGGACCGTAACAGCCGTACAATTGGTAACACTCATCTTTAAAGGACATACAGGGGAGATGTTTCTTCAG

215 E Y W T V T A V Q L V T L I F K G H T G E M F L Q

1801 AATGTGACTGACATTGAATCTGTGACTTTTATCATGGAGCAGTCGGAGGACTCTGATCGTGAATGTCTGATGTGT

240 N V T D I E S V T F I M E Q S E D S D R E C L M C

1876 GGTTACCAGCACAGTGACAAAGACTTTCAGTGCATTAATTACCTCGAGGAAAGTATCTCTAAAAATCTGCGACTA

265 G Y Q H S D K D F Q C I N Y L E E S I S K N L R L

1951 CGGCATGGATCTAGCCAGGACAGCCGCAGTTCCTCCCCGAACGGCTCAAGTCTGGAGGTCTCCAGTCAGGAACAG

290 R H G S S Q D S R S S S P N G S S L E V S S Q E Q

2026 GAGAAATTACATAAATATTTGGAAGTTTTTGCTGTACAGTTTCTGACCAAAGCTTTCTGTAACACGGTTCATGTT

315 E K L H K Y L E V F A V Q F L T K A F C N T V H V

2101 CTCAGGAATATTCTCACATCTCAATACAAAGCAATGATAGATGAAGTGTATATACTATGGCTCCTGGCATTTTTT

340 L R N I L T S Q Y K A M I D E V Y I L W L L A F F

2176 CTGAAATATGCGCGACATCAGCGAGTGGAATTCCATTACATCAGGGGAATCTTCTCCACAGACATGTTTGCATTT

365 L K Y A R H Q R V E F H Y I R G I F S T D M F A F

2251 CTGGTTTACAAAACAGTGGTGAATTCTGAGGAGCTAGTGGTAGCACAGAGAAACAAACAGGACACCACTCATCAC

390 L V Y K T V V N S E E L V V A Q R N K Q D T T H H

2326 CTCAGAAGAATTCATCTGACTGTTGGTGCTATGCGGGAAATGTTACAAGGTCTGAAGCTATATTGCGAAGATCAG

415 L R R I H L T V G A M R E M L Q G L K L Y C E D Q

2401 AGTCTAGATAAAGCCAACATAGCTTACCTAGAGAATTTACAAAAAGACTTGGCCAACTCTGTAGACCTGCCCCAG

440 S L D K A N I A Y L E N L Q K D L A N S V D L P Q

2476 ATGTTCCTCCTCCTGATCCGGAACTTCCAGATGCACACACACAGTCTGAGATTCCTCCAGGACGTGATCATCACC

465 M F L L L I R N F Q M H T H S L R F L Q D V I I T

2551 AACCACCTGCTGCTACTGCTGGTGGACGTGTGGAGGTCCCAGTCCATCAGCAAGACAGAGTTCCACATGCTGAAC

490 N H L L L L L V D V W R S Q S I S K T E F H M L N

2626 CACATCAAACAATTTGCCACAACAGAAGTGATGCAGAAGTATGGCCGCTTGTTAGAGAACTTTGAGTATAACAAT

515 H I K Q F A T T E V M Q K Y G R L L E N F E Y N N

2701 GCCGTAGTAAACAACTGTATATTTACCATGATGCACCATGTTGCTGGAGATTGCGAGAAACCCAATGCCTTGCTG

540 A V V N N C I F T M M H H V A G D C E K P N A L L

2776 CAGGTGCAAATATTGCGGACCTTCCTAGAGATTTTGGACAGCAACGCCCCGTTAACTCAGGAGAAGACAGACTTG

565 Q V Q I L R T F L E I L D S N A P L T Q E K T D L

2851 ATTGAGTTCATCCTACAGAAATTCATGGTTGAGGCAGAGAACAATCCATTTTCCTGTGCTCTACAGCTGTTTGGG

590 I E F I L Q K F M V E A E N N P F S C A L Q L F G

2926 GAAATGCCAGTCAAATCTAAACATCGGAAAACTGAAGAGGAGGAGGATGAGGTTGCAGATGATGCTGAAGATGAA

615 E M P V K S K H R K T E E E E D E V A D D A E D E

3001 GAAGAGGATGAGAATGCTGAAGAAATGAAGAAGGAGAAGGACATTCTTCTGATGCTGTACACGGAGTTGATGGGA

640 E E D E N A E E M K K E K D I L L M L Y T E L M G

3076 CAGCCCGGGGCTCTTACTGCTATCTCCACCAAACTGGGCGAGTTTGGGATCAAGAAAACAGAGCAACAGATTTCT

665 Q P G A L T A I S T K L G E F G I K K T E Q Q I S

3151 GAAGACCTCAAAGAATTTGATTGGTTGGTCGATGAAAGAAACACAGAAGGGTCAGAGAAGACGAGATCTGTGGAA

690 E D L K E F D W L V D E R N T E G S E K T R S V E

3226 TCGGACATGGAACAGAGCAACTCCTCCTCGTCCTTCGCTGATGACTTAGAAGACCTAGATGACTCGGAGATAGTC

715 S D M E Q S N S S S S F A D D L E D L D D S E I V

3301 ACTTACTGTATCACGAAGCTAAAGGAAATGAAGTGTGAGGACCATCTGAAGTGGCTCCAACAACAGTTCTGTGAT

740 T Y C I T K L K E M K C E D H L K W L Q Q Q F C D

3376 GTTGCCTATGCCAAGTGTGCAATGTCTATGAGTAGTTTCGACAAAAATGAAACAGAAGGATTTGTAGCCAGATTA

765 V A Y A K C A M S M S S F D K N E T E G F V A R L

3451 CACGTCATTCAGAATAAGTCTGTACCGCTGATTCTCTACAACGAAGAACAGGAACAACTGATGCACAACCCGTAC

790 H V I Q N K S V P L I L Y N E E Q E Q L M H N P Y

3526 TTTATCACCCTGCTTCAGTTCCTGGGGTTACACATGCCAGAGGATGTGGGATTGGTGTTTCCCAGAATTCCTCAC

815 F I T L L Q F L G L H M P E D V G L V F P R I P H

3601 TTCTGGACCATTCAGAATCTGGTTGAGAAGGCTAGCCAGTTTGGTGATCTCACCATTGATCAACTAAAGTTTGAC

840 F W T I Q N L V E K A S Q F G D L T I D Q L K F D

3676 CCAGTGAAGTTGAAAGAAGGAAGGGAAATGACGGAGATAATGCTGCCCTTCAAACTGGAGAAAAAGGAGGACATC

865 P V K L K E G R E M T E I M L P F K L E K K E D I

3751 ACATTTGAGAAAATGAAAAAGATCCCGGACACAGTATGGATAACAATGATCCAGCAATACAACAAAAATTCTGGA

890 T F E K M K K I P D T V W I T M I Q Q Y N K N S G

3826 AAGGATGGGAAACAAGGAATGCAAGTCAGGAACGAGTTCCAGAGCCAGGCTTGTCCT**TGA**ccttcaacccattct

915 K D G K Q G M Q V R N E F Q S Q A C P *

3901 gactgagtccatatcggggaggacggcccattactcgctgaattgtcaaaggaaatgctgcaaaatgctggcatt

3976 gaacagtgtttccataagtgctggaaacatttatttgatattttcattgtgtgtgaatccctttggaaaacttgg

4051 atactttcgtgaagacaatctctgcagagaaaaacttggacgtgaattaaaacacttgtgtcattgtgaatttaa

4126 tctgcagatttatttccctgtgtacaatatatgtacagttttagttatacaagtacaaaaaaggaaatatatacc

4201 atgattgctccaaatgagacagcaccaaacttttagcaaatcatgaaatattcgcaacccagtgtaatttgtaca

4276 gcaattgaacaatgtggtacatgtaattatggtagctctataatgattatgtaagatagagatggaccatgctgt

4351 atcttctgaattgtaaatgatttgaagtgattgagcattctttgttttgaagaatgtgcccctgatcggtaatct

4426 ttcctacctggagaagctgtagaccatgtggtcatttacctagtatttttcatctttttgtcttcatctccacac

4501 agacttcagtattgtacagtatttaaatgtgataatcgtttcatttaatgttgtcttctctggtagttactccca

4576 aaaagcttggggtaaaaaattatcgttggtcatctagtgccaattttacttcgtttatgtattttagagctcagt

4651 tgaaaaaaatatgatcaataaatcttgttcaactgaatatagtgtgcaatttcttaaacatttatgtgtaaataa

4726 aacaatcgcacaaatcaacactggttttaacagaatttaatgtatactatttatatagtttcggttttaaatata

4801 ttgctgaaaatttattactgagcatgtttgcatcaggttacgtgtatatattacaggtaatgccactgtagtaaa

4876 atcatcaaatacatgtaaatgtatttattttgttgtaacttaattgaatggtttttaataaatttgttgaaatgt

4951 aaaaaaaaaaaaaaaaa

>CgP-likeCry

1 aggagttccggcacatattatgtaaacataaatttttcacgttgtcgttgataacatttgggt**ATG**GAGTCCCCT

1 M E S P

76 ACTAGAAAAGAAATCTTGCATAATTTTCAGACAGGGGTCATCGACGCTGAAGAATGTTTCTGTATGATTGTTTCT

5 T R K E I L H N F Q T G V I D A E E C F C M I V S

151 CTCGATGGTTATGATTCAACGAGACAACATTTCCTGAAAAACATTGATTTCTTGAGGCTCACTAATCCTAAGAAG

30 L D G Y D S T R Q H F L K N I D F L R L T N P K K

226 TACAACGAACTCTTCACCGTTTTTGCAAATTACTTTGAAAAACCTCCATCTGGTGCATTTTTAGATCCTATAGGT

55 Y N E L F T V F A N Y F E K P P S G A F L D P I G

301 TGTAGTGTTGAAAATGGATACTTAACAAGTGACTTTGGGTATGAACTGGAAGTAGAACTCGCATCAGCCCTGTCA

80 C S V E N G Y L T S D F G Y E L E V E L A S A L S

376 TTGCAAGATCAGAGTGAAAGCCTTAACAGCAAAGCATCTTATGTTAGTGTTGTACAGGAGCCAATTAGTAAACAA

105 L Q D Q S E S L N S K A S Y V S V V Q E P I S K Q

451 AGAAATACATCAAGTAATTCTGAAAGAAATCAACCAAAGAATGTTAACAATAGTTACTCAAAAAATGTGAAAGAT

130 R N T S S N S E R N Q P K N V N N S Y S K N V K D

526 GACAATGATTCAGTTACTTCATCGGTCAAAAGTAAAAAAAGGAAAAATAAAGGCTCCACAAACAGGCCTGTGGTT

155 D N D S V T S S V K S K K R K N K G S T N R P V V

601 TACTGGTTCAGGAGAGATCTTCGTCTGTATGACAATCCAGCATTGTTTGAAGCGGCCAGTATGAATGTCCCCGTT

180 Y W F R R D L R L Y D N P A L F E A A S M N V P V

676 ATATTGGTTTTCTTGTGGTCAGAATCAGAGGAGGATCCAGAGGGTGTGGTGGCAGCAGGAGGAGCCACTAAACTA

205 I L V F L W S E S E E D P E G V V A A G G A T K L

751 TGGTTACACCACGCTCTGAACCACCTGGACAAATCCATCAGTGACAGATACAACAACAGGATCATCTACAGGAAA

230 W L H H A L N H L D K S I S D R Y N N R I I Y R K

826 ACCCAGTCCTGTCAAAGAGAAATCCTGTCACTTATTGAAGAAACTGGTGCCAAAGCATTGCTGATAAATGATGTC

255 T Q S C Q R E I L S L I E E T G A K A L L I N D V

901 TATGAACCATTCCTCAAACAAAGAGATGACAAGATTTGTTCGGAACTACAGAGGAAAGGGATAGAATGTAAGAGA

280 Y E P F L K Q R D D K I C S E L Q R K G I E C K R

976 TTCCACTCTTACTTGCTGCATGAGCCAGGTTCTGTGTCAGCGGAGTCTGTGGGGATGAGAGGGGTGGGCTCAGTG

305 F H S Y L L H E P G S V S A E S V G M R G V G S V

1051 ACCCACTTTATGGAGTGCTGTAGGCAATCTGATGCCCAGCCCATTGGGCATCCTCTGGATTATCCCCCCACTCTA

330 T H F M E C C R Q S D A Q P I G H P L D Y P P T L

1126 CCCAAACCAGACCAGTTTCCCTCCAGCTCATCTCTTCATGATCTAGAACTAGCCAAAATGCCAAGGAGAAAAGAT

355 P K P D Q F P S S S S L H D L E L A K M P R R K D

1201 GGATCTATTATTGACTGGGCAGCACCAATTGTGAGACAGTGGGATTTTGGAGAAGAAGGAGCGTGGAAAGCTCTA

380 G S I I D W A A P I V R Q W D F G E E G A W K A L

1276 GAACTTTTCTTATCTGAAGGTGTCAGGAAATATGAGAAGGAGTCGTGTCGCACCGACCACCTGAACACCTGTCGG

405 E L F L S E G V R K Y E K E S C R T D H L N T C R

1351 ATCTCTCCGTACCTTCACTTCGGTCAGATCAGTCCCCGGGCGGTGCTGGAGGAAGCCAGACACATGAAGTCGCCC

430 I S P Y L H F G Q I S P R A V L E E A R H M K S P

1426 AAGTTCCTCCGGAAGCTGGCCTGGAGAGACCTGTCTTACTGGCTGCTGACCCTCTGGCCTGACCTCCCCTCACAG

455 K F L R K L A W R D L S Y W L L T L W P D L P S Q

1501 CCCACCAGGGTGCATTACAGGGATCAAGCCTGGAGCAGGGACGCTGGTCACTTGAAGGCATGGCAGAGAGGTAGG

480 P T R V H Y R D Q A W S R D A G H L K A W Q R G R

1576 ACGGGGTTCCCCCTGGTGGATGCCGCGATGAGACAGCTGTGGCTGGAGGGGTGGATCAACAACTACCTCAGACAC

505 T G F P L V D A A M R Q L W L E G W I N N Y L R H

1651 GTGGTCGCCTCCTTCCTCATCTCCTACCTCCGCCTCCACTGGGTGGAGGGCTACCGCTGGTTCCAGGACACCTTA

530 V V A S F L I S Y L R L H W V E G Y R W F Q D T L

1726 CTGGATGCTGATGTGGCCATCAATGCCATGATGTGGCAGAATGGAGGAATGAGTGGGCTGGACCAGTGGAACTTT

555 L D A D V A I N A M M W Q N G G M S G L D Q W N F

1801 GTAATGCACCCCGTGGATGCAGCTTTGACCTGTGACCCCGATGGAGCCTATGTCAGAAAGTGGTGTCCAGAGATA

580 V M H P V D A A L T C D P D G A Y V R K W C P E I

1876 GCAGCCCTCCCCAACGACTTCATACATCAGCCCTGGAAATGTCCACCCTCCATTCTGAGGCGATGTGGTATAAAG

605 A A L P N D F I H Q P W K C P P S I L R R C G I K

1951 TTAGGAGAGACATACCCAAATCGGGTTATATCAGATTTGGAGGGAGCAAGGGAGCAGTCATTAACGGATGTTGTA

630 L G E T Y P N R V I S D L E G A R E Q S L T D V V

2026 AATGTTCGGAAGAAGCACCCAGAGTTTGTGGACCGCCGAACGGGCAACGACCTTGTGCCCCTCCCTGATGGTCTG

655 N V R K K H P E F V D R R T G N D L V P L P D G L

2101 TGTGTCCCGGTCATCACCAGGAAGGAGTTCAAGTACAAGTTGCACCACCCAGAGGCCAAGGACAACCCCCACACA

680 C V P V I T R K E F K Y K L H H P E A K D N P H T

2176 GCTGTACTGAGGGGGTACCGGTCCAGGAAGAGGGACGAAGCCATTGCTTTTGCCAACGAGAGAGACTTCATGGCT

705 A V L R G Y R S R K R D E A I A F A N E R D F M A

2251 AGTGCTATGAATGAAAGTGTGAAGCTTAGTGAGAGAAGATTAAAGGCAACGCAATATGAAGCACTC**TGA**tgaagt

730 S A M N E S V K L S E R R L K A T Q Y E A L *

2326 agtgtgtagaatttgcaaatatcgggtgtacgtgagctgtttagtgttggaggattgaggacatgagtgtgtgat

2401 acgcataaatgaagatgttgtcttgggaaatggtttggaatatgtgaagaatggggtgttttctctgacaataat

2476 gacttgaacacatgtgtgtggcaactgccttcttctttgtagcagtgcatgtggttaaggaggaagtacatgtat

2551 tt
