# Supplementary material for: Identification of the Molecular Clockwork of the Oyster Crassostrea gigas
Source: PLoS One. 2017 Jan 10;12(1):e0169790. doi: 10.1371/journal.pone.0169790 (PMC5224872; doi:10.1371/journal.pone.0169790)
Supplement: S1 Table — (DOC) [file pone.0169790.s003.doc]

Table S1. Nucleotide sequences of specific primer pairs used in the present study.

|  | Primer names | Primer sequences |
| --- | --- | --- |
| RACE |  |  |
|  | Qt | CCAGTGAGCAGAGTGACGAGGACTC |
|  |  | GAGCTCAAGCTTTTTTTTTTTTTTTTT |
|  | Qo | CCAGTGAGCAGAGTGACG |
|  | Qi | GAGGACTCGAGCTCAAGC |
|  | Cg6-4photolyaseRT | GGGTAGTCTTTCCCAACAATACA |
|  | Cg6-4photolyaseRo | CTGTTGGGTACTTACTGAGCTG |
|  | Cg6-4photolyaseRi | AATACTGGCTTCACGTCGTC |
|  | Cg6-4photolyaseFo | TTATGAGCCATGGAAGGCAC |
|  | Cg6-4photolyaseFi | TGTATTGTTGGGAAAGACTACCC |
|  | CgClockRT | CTCGACGGAAGTGACAGG |
|  | CgClockRo | TTGATTGGTGAGGTCGGC |
|  | CgClockRi | GCAACTGTCCAATGCCTC |
|  | CgClockFo | TGTGGAGGAACTTCCGTC |
|  | CgClockFi | TTACTTCTCCAGCAGCGC |
|  | CgCry2RT | GGTTTCCACGGTCAGTGT |
|  | CgCry2Ro | CAACATGGCTACCCGTGA |
|  | CgCry2Ri | GGATGGTTCCCAGAATGC |
|  | CgCry2Fo | TCACGGGTAGCCATGTTG |
|  | CgCry2Fi | ACACTGACCGTGGAAACC |
|  | CgBmalRT | GCTGGTGGTCTCCCAATC GATTGGGAGACCACCAGC ok |
|  | CgBmalRo | GCCTGTCATTGCCTGACT |
|  | CgBmalRi | GCTGGTGGTCTCCCAATC |
|  | CgBmalFo | AGTCAGGCAATGACAGGC |
|  | CgBmalFi | GTTATCCTCCATGCTGCCC |
|  | CgPeriodRT | CTCAGAGGAGGAGAAGGATG |
|  | CgPeriodRo | GAAGCCACTATGCTGTAACC |
|  | CgPeriodRi | GGTTTGCTCTCAACTGGAG |
|  | CgPeriodFo | GACAGCTGGATCAACAGAAG |
|  | CgPeriodFi | CTCCAGTTGAGAGCAAACC |
|  | CgTimelessRT | TCCGAGTCATCTAGGTCTTC |
|  | CgTimelessRo | GAAGGACGAGGAGGAGTT |
|  | CgTimelessRi | CGTCTTCTCTGACCCTTCT |
|  | CgTimelessFo | GAAGAGGAGGAGGATGAGG |
|  | CgTimelessFi | CTGCTATCTCCACCAAACTG |
|  | CgPlikeCryRT | GGCTCCTGTACAACACTAAC |
|  | CgPlikeCryRo | GGCTGATGCGAGTTCTAC |
|  | CgPlikeCryRi | AAATGCACCAGATGGAGG |
|  | CgPlikeCryFo | GGAAGTAGAACTCGCATCAG |
|  | CgPlikeCryFi | GTTAGTGTTGTACAGGAGCC |
|  | CgCry1RT | CTCTCCAGTATGTCAGTGGG |
|  | CgCry1Ro | CTATCGCACTCGGAGAGTC |
|  | CgCry1Ri | TCTATCAAGGACGGGTTGTC |
|  | CgCry1Fo | TCCTCCAATGCCCTAACTG |
|  | CgCry1Fi | CACTTCCTGTCCAGCAGA |

|  | Primer names | Primer sequences |
| --- | --- | --- |
| Quantitative real-time PCR | | |
|  | Cg6-4photolyase-F | CAGCTCAGTAAGTACCCAACAG |
|  | Cg6-4photolyase-R | GGGTAGTCTTTCCCAACAATACA |
|  | CgClock-F | CGCTACTACATAACGTACCATCAG |
|  | CgClock-R | TGTTCTCTAACATCGGCATAGC |
|  | CgCry2-F | AACCTTACAGCAAGCACGAA |
|  | CgCry2-R | TGACATCTGGCTGTGGTTTC |
|  | CgBmal-F | CACAAGTTCAGGTCAGAGTGTAG |
|  | CgBmal-R | TCACCTGAGGTAGACTGGTTAT |
|  | CgPeriod-F | CCGATGACAGAAATCCCAGTAG |
|  | CgPeriod-R | CCATCCTATTCTCCTGCTCTTG |
|  | CgTimeless-F | AAAGATCCCGGACACAGTATG |
|  | CgTimeless-R | TGGAACTCGTTCCTGACTTG |
|  | CgPlikeCry-F | CCAGGAAGGAGTTCAAGTACAA |
|  | CgPlikeCry-R | CAATGGCTTCGTCCCTCTT |
|  | CgCry1-F | TCATGAAGCAGCTCAGATACG |
|  | CgCry1-R | ACCTCCCAGTTCAACCAAAG |
|  | CgRev-Erb-F | GACTTTGCTGATCGCTTCAAC |
|  | CgRev-Erb-R | CTTTCCAACTGCTCCACATTTC |
|  | CgROR-F | CTACGTGAGCAGGTGTTTGA |
|  | CgROR-R | CGTCCGCTATGTCCTTCAAT |
|  | EF1-F | ACCACCCTGGTGAGATCAAG |
|  | EF1-R | ACGACGATCGCATTTCTCTT |

Elongation factor 1 (*EF1*, AB122066) was used as housekeeping genes. F and R designate forward, and reverse primers respectively.
