# Supplementary material for: Identification of the Molecular Clockwork of the Oyster Crassostrea gigas
Source: PLoS One. 2017 Jan 10;12(1):e0169790. doi: 10.1371/journal.pone.0169790 (PMC5224872; doi:10.1371/journal.pone.0169790)
Supplement: S2 Table — (DOC) [file pone.0169790.s004.doc]

Table S2.

| Species | Designation | Accession Number |
| --- | --- | --- |
| *Acropora millepora* | Cry 1 | ABP97098.1 |
| *Acropora millepora* | Cry 2 | ABP97099.1 |
| *Adiantum capillus-veneris* | Cry 1 | BAA88423 |
| *Aedes aegypti* | 6-4 photolyase | XP_001658195 |
| *Aedes aegypti* | clock | XP_001662706.1 |
| *Aedes aegypti* | CRY1 | XP_001648498 |
| *Aedes aegypti* | CRY2 | XP_001655778 |
| *Aedes aegypti* | cycle | AEX32872.1 |
| *Aedes aegypti* | period | XP_001658976.1 |
| *Aedes aegypti* | timeless | AAY40757.1 |
| *Aedes aegypti* | timeout | XP_001660177.1 |
| *Anopheles darlingi* | 6-4 photolyase | ETN57794.1 |
| *Anopheles darlingi* | 6-4 photolyase2 | ETN59907.1 |
| *Anopheles gambiae* | Cry 2 | ABB29887.1 |
| *Apis mellifera* | Cry 2 | NP_001077099.1 |
| *Aplysia californica* | period | XP_005111020.1 |
| *Aplysia californica* | timeless | XP_005099695.1 |
| *Arabidopsis thaliana* | Cry 1 | NP_567341 |
| *Arabidopsis thaliana* | Cry_DASH | NP_568461 |
| *Astyanax mexicanus* | Cry1 | AHA91700.1 |
| *Bombyx mori* | Cry 1 | NP_001182628.1 |
| *Bombyx mori* | Cry 2 | NP_001182627.1 |
| *Bombyx mori* | timeless | NP_001037622.1 |
| *Branchiostoma floridae* | bmal | AGX25233.1 |
| *Branchiostoma floridae* | clock | AGX25232.1 |
| *Branchiostoma floridae* | period | EEN70104.1 |
| *Bulla gouldiana* | period | AAK97374.1 |
| *Culex quinquefasciatus* | Cry 1 | XP_001867895.1 |
| *Culex quinquefasciatus* | Cry 2 | XP_001869456.1 |
| *Danaus plexippus* | 6-4 photolyase | ABO38436 |
| *Danaus plexippus* | clock | AAR13011.1 |
| *Danaus plexippus* | CRY1 | AAX58599 |
| *Danaus plexippus* | CRY2 | ABA62409 |
| *Danaus plexippus* | cycle | EHJ64590.1 |
| *Danaus plexippus* | period1 | AAO48719.1 |
| *Danaus plexippus* | timeless | EHJ67997.1 |
| *Danaus plexippus* | timeout | EHJ76705.1 |
| *Danio rerio* | 6-4 Photolyase | NP_571863 |
| *Danio rerio* | bmal | AAF64395.1 |
| *Danio rerio* | clock | NP_571032.1 |
| *Danio rerio* | Cry_DASH | Q4KML2 |
| *Danio rerio* | CRY2a | BAA96846 |
| *Danio rerio* | CRY2b | BAA96850 |
| Species | Designation | Accession Number |
| *Danio rerio* | period1 | NP_997604.2 |
| *Danio rerio* | period2 | NP_878277.1 |
| *Danio rerio* | period3 | NP_571659.1 |
| *Danio rerio* | P-like | XP_009291670.1 |
| *Danio rerio* | timeless | NP_001265529.1 |
| *Daphnia pulex* | 6-4 photolyase | EFX85418.1 |
| *Daphnia pulex* | Cry 2 | EFX82092.1 |
| *Drosophila melanogaster* | 6-4 Photolyase | BAA12067 |
| *Drosophila melanogaster* | clock | O61735.3 |
| *Drosophila melanogaster* | CRY1 | NP_732407 |
| *Drosophila melanogaster* | cycle | AAF49107.1 |
| *Drosophila melanogaster* | period | AAF45804.1 |
| *Drosophila melanogaster* | timeless | P49021.3 |
| *Drosophila melanogaster* | timeout | AAF54908.3 |
| *Euphausia superba* | Cry | CAQ86665.1 |
| *Euprymna scolopes* | Cry 1 | AGJ94014.1 |
| *Euprymna scolopes* | Cry 2 | AGJ94015.1 |
| *Eurydice pulchra* | 6-4 photolyase | AGV28718.1 |
| *Eurydice pulchra* | bmal | AGV28715.1 |
| *Eurydice pulchra* | clock | AGV28721.1 |
| *Eurydice pulchra* | Cry 2 | AGV28717.1 |
| *Eurydice pulchra* | period | AGV28714.1 |
| *Eurydice pulchra* | timeless | AGV28716.1 |
| *Homo sapiens* | bmal | BAA19935.1 |
| *Homo sapiens* | clock | AAB83969.1 |
| *Homo sapiens* | CRY2a | NP_004066 |
| *Homo sapiens* | CRY2b | AAH41814 |
| *Homo sapiens* | period1 | O15534.2 |
| *Homo sapiens* | period2 | O15055.2 |
| *Homo sapiens* | period3 | P56645.4 |
| *Homo sapiens* | timeless | Q9UNS1.2 |
| *Nematostella vectensis* | Cry_DASH | XP_001623243 |
| *Oryza sativa* | Cry 1 | BAB70686 |
| *Oryza sativa* | Cry_DASH | Q651U1 |
| *Oryzas latipes* | Cry_DASH | XP_004081133 |
| *Pacifastacus leniusculus* | bmal | AFV39705.1 |
| *Pacifastacus leniusculus* | clock | AFV39704.1 |
| *Platynereis dumerilii* | bmal | AGX93014.1 |
| *Platynereis dumerilii* | clock | AGX93013.1 |
| *Platynereis dumerilii* | CPD | AIE57497.1 |
| *Platynereis dumerilii* | Cry | AIE45779.1 |
| *Platynereis dumerilii* | Cry 2 | AGX93012.1 |
| *Platynereis dumerilii* | Cry DASH | AIE57496.1 |
| *Platynereis dumerilii* | CRY1 | AEJ87227 |
| *Platynereis dumerilii* | period | AEJ87229.1 |
| Species | Designation | Accession Number |
| *Platynereis dumerilii* | timeless | AGX93010.1 |
| *Rattus norvegicus* | bmal | BAA33450.1 |
| *Rattus norvegicus* | clock | NP_068628.1 |
| *Rattus norvegicus* | CRY2a | NP_942045 |
| *Rattus norvegicus* | CRY2b | EDL79575 |
| *Rattus norvegicus* | period1 | Q8CHI5.2 |
| *Rattus norvegicus* | period2 | Q9Z301.1 |
| *Rattus norvegicus* | period3 | Q8CJE2.1 |
| *Rattus norvegicus* | timeless | Q9Z2Y1.1 |
| *Solanum lycopersicum* | Cry 1 | NP_001234667 |
| *Solenopsis invicta* | clock | AGD94516.1 |
| *Solenopsis invicta* | Cry | AGD94517.1 |
| *Solenopsis invicta* | cycle | AGD94518.1 |
| *Strongylocentrotus purpuratus* | Cry_DASH | XP_783613 |
| *Takifugu rubipres* | Cry_DASH | XP_003968037 |
| *Thermobia domestica* | clock | BAJ16353.1 |
| *Thermobia domestica* | cycle | BAJ16354.1 |
| *Thermobia domestica* | timeless | BAL27710.1 |
| *Tribolium castaneum* | clock | NP_001106937.1 |
| *Tribolium castaneum* | Cry 2 | NP_001076794.1 |
| *Tribolium castaneum* | cycle | NP_001107795.1 |
| *Tribolium castaneum* | period | NP_001106933.1 |
| *Tribolium castaneum* | timeless | EFA04644.2 |
| *Schizosaccharomyces pombe* | Swi1 | Q9UUM2.1 |
| *Xenopus laevis* | 6-4 Photolyase | NP_001081421 |
| *Xenopus laevis* | bmal | AAW80970.1 |
| *Xenopus laevis* | clock | AAF34772.1 |
| *Xenopus laevis* | Cry_DASH | NP_001084438 |
| *Xenopus laevis* | CRY2a | AAK94665 |
| *Xenopus laevis* | CRY2b | AAK94666 |
| *Xenopus laevis* | period1 | NP_001079172.2 |
| *Xenopus laevis* | period2 | NP_001081098.1 |
| *Xenopus laevis* | timeless | BAE45344.1 |
| *Crassostrea gigas* | HIF | BAG85183 |
| *Crassostrea gigas* | NPas | EKC18855 |
| *Crassostrea gigas* | Cry 1 | KT991835 |
| *Crassostrea gigas* | Cry 2 | **KX371074** |
| *Crassostrea gigas* | bmal | **KX371075** |
| *Crassostrea gigas* | 6-4 Photolyase | **KX371078** |
| *Crassostrea gigas* | clock | **KX371073** |
| *Crassostrea gigas* | timeless | **KX371077** |
| *Crassostrea gigas* | Period | **KX371076** |
| *Crassostrea gigas* | P-like cry | **KX371079** |
